# Use of Western Medicine and Traditional Korean Medicine for Joint Disorders: A Retrospective Comparative Analysis Based on Korean Nationwide Insurance Data

**DOI:** 10.1155/2017/2038095

**Published:** 2017-12-06

**Authors:** Boyoung Jung, Sukjin Bae, Soyoon Kim

**Affiliations:** ^1^Department of Public Health, Graduate School, Yonsei University, 50 Yonsei-ro, Seodaemun-gu, Seoul 03722, Republic of Korea; ^2^Research Department, Research Institute of Korean Medicine Policy, 91 Heojun-ro, Gangseo-gu, Seoul 07525, Republic of Korea; ^3^Asian Institute for Bioethics and Health Law (WHO Collaborating Centre for Health Law and Bioethics), Yonsei University, 50 Yonsei-ro, Seodaemun-gu, Seoul 03722, Republic of Korea; ^4^Department of International Health, Graduate School of Public Health, Yonsei University, 50 Yonsei-ro, Seodaemun-gu, Seoul 03722, Republic of Korea

## Abstract

This study aimed to compare the usage of Western medicine and traditional Korean medicine for treating joint disorders in Korea. Data of claims from all medical institutions with billing statements filed to HIRA from 2011 to 2014 for the four most frequent joint disorders were used for the analysis. Data from a total of 1,100,018 patients who received medical services from 2011 to 2014 were analyzed. Descriptive statistics are presented as type of care and hospital type. All statistical analyses were performed using IBM SPSS for Windows version 21. Of the 1,100,018 patients with joint disorders, 456,642 (41.5%) were males and 643,376 (58.5%) were females. Per diem costs of hospitalization in Western medicine clinics and traditional Korean medicine clinics were approximately 160,000 KRW and 50,000 KRW, respectively. Among costs associated with Western medicine, physiotherapy cost had the largest proportion (28.78%). Among costs associated with traditional Korean medicine, procedural costs and treatment accounted for more than 70%, followed by doctors' fees (21.54%). There were distinct differences in patterns of medical care use and cost of joint disorders at the national level in Korea. This study is expected to contribute to management decisions for musculoskeletal disease involving joint disorders.

## 1. Introduction

Musculoskeletal diseases are one of the leading causes of disability worldwide. It is a major contributor to health burden and health care costs [1]. Korea has a rapidly aging population due to the decrease in birth rate and increase in life expectancy. The percentage of the population aged 65 years or older will increase from 10.3% in 2008 to 15.6% in 2020 and 38.2% in 2050 [2]. Previous studies have reported findings on the prevalence of musculoskeletal disease [3, 4]. As the Korean population continues to age, the economic burden of musculoskeletal disease will continue to increase. The total economic burden of treating musculoskeletal is about 8.1 billion dollars [2]. Musculoskeletal diseases are the most common health problems that require the use of traditional Korean medicine [5], an integral part of prevailing practice and belief systems throughout Korea's history. Starting from the late 19th century, Western medical practices were introduced by Christian missionaries to Korea. These practices quickly supplanted traditional Korean medicine in institutional health care. After the Korean War, the government revived interest in traditional Korean medicine and established colleges that specialize in that field, in addition to colleges of Western medicine [6]. With this historical background, the Korean medical system is characterized by a dual [7, 8], mutually exclusive medical system consisting of Western medicine and traditional Korean medicine practices [9]. In Korea, primary care physicians work mostly in solo private practices and are reimbursed on a fee-for-service basis. This system enables patients to choose and retain individual physicians regardless of changes in employment status. Therefore, Koreans can use both Western and traditional Korean medicine to treat musculoskeletal disorders. Previous studies have assessed the prevalence and cost of Korean medicine [5, 6, 10–13]. However, most of these studies did not focus on joint disorders [11, 14–16]. Particularly, the statuses of health care utilization associated with joint disorders including the scale of the whole population and health care costs of patients receiving treatment for joint disorders are currently unknown. Therefore, the objective of this study was to analyze claim data submitted to the Korean National Health Insurance (NHI) and assessed by the Health Insurance Review and Assessment Service (HIRA) to compare medical care use between Western medicine and traditional Korean medicine. The results of this study will provide basic information for future management decisions for musculoskeletal diseases especially joint disorders in Korea.

## 2. Materials and Methods

### 2.1. Data Source

This study used claims data from the 2011–2014 National Patient Sample (NPS) dataset of HIRA. Datasets generated and/or analyzed for this study period are available from the HIRA-NPS repository [17]. The NPS includes 3% sample data of 2011–2014 national insurance billing data. It can represent the country as a whole (46 million patients). The total number of filed claims and total health expenditure have increased steadily. As of 2011, the total number of filed claims has reached 1.3 billion, with a total health expenditure of ~51.5 trillion KRW. Patients were stratified according to sex and age in 5-year intervals. These HIRA claim data are compiled by health care providers nationwide. They correspond to the number of claims submitted by patients. Claims from patients with the Medicaid program, government expenditures, and veteran patients are also included in these claim data [18]. All data were deidentified to ensure patient confidentiality. The HIRA Research Ethics Committee of South Korea approved the study protocol.

### 2.2. Study Population

**Figure 1:**
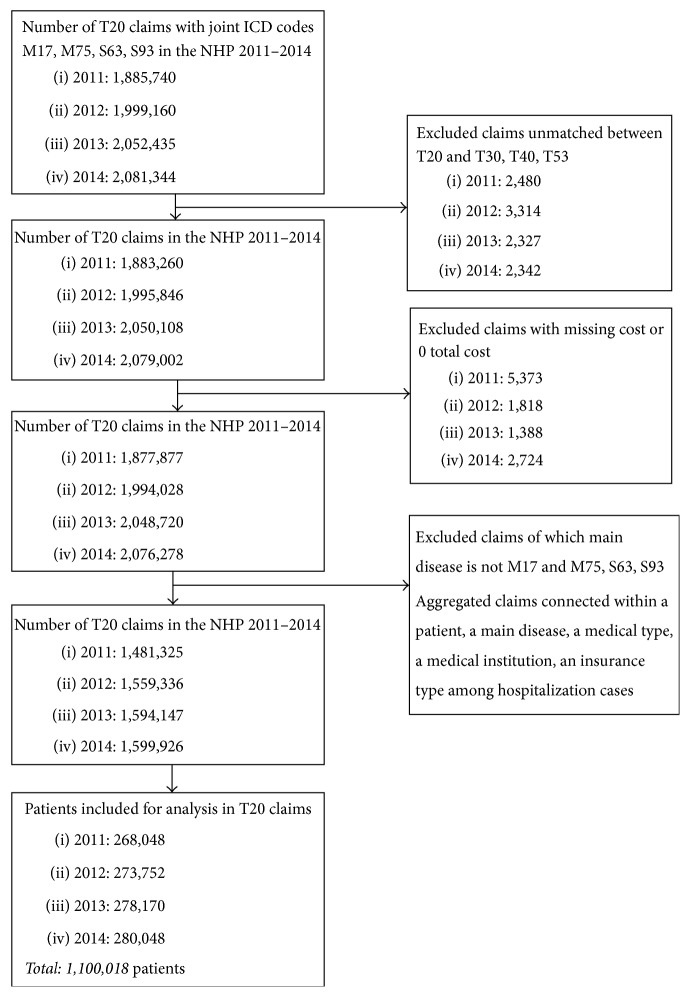
Flowchart of the study sample.

After reviewing frequent diseases each year in traditional Korean medicine as described previously [19], patients with the following four most frequent joint disorders were included in this study: M17 (gonarthrosis [arthrosis of knee]), M75 (shoulder lesions), S63 (dislocation, sprain, and strain of joints and ligaments at wrist and hand level), and S93 (dislocation, sprain, and strain of joints and ligaments at ankle and foot level). Although dorsalgia (M54) was at the top of the list, it was excluded from analysis since there was no change in ranking by year. To observe changes in the ranking of diseases by year, the remaining joints were included in the study. We focused on musculoskeletal disorders and injuries of the extremities. The diagnoses were coded using the 6th revision of the Korean Classification of Diseases (KCD-6) adapted from the International Classification of Diseases, 10th revision. Data from the billing statements for patients with missing cost data and those with zero total cost were excluded. Patient might have visited more than once during the study period (i.e., more than one claim per patient). Therefore, the number of claims in this study was higher than the total number of patients. A total of 7,996,903 claims for 1,100,018 patients with joint diseases with prefix codes of M17, M75, S63, and S93 in primary diagnoses were included for analysis through discussion of a panel of three clinicians (one public health specialist, one Korean medical doctor, and one statistician). A total of 1,100,018 patients were finally included in our analysis (Figure 1).

### 2.3. Episode Creating Process

Claims data provided by HIRA included raw data of treatment prescriptions for all patients who received medical services over the course of one year after removing personal and corporate information. Because the claims were submitted monthly, charges in the statement reflected up to one month of information. In other words, patients who had been hospitalized for more than one month would have been charged separately for each month. In such cases, errors such as overestimation of the number of inpatients and underestimation of medical expenses might occur when performing statistical analyses. Therefore, episodes, involving collecting and calibrating several claims charged monthly for one consecutive medical practice were used. In this study, separated claim forms of hospitalized patients were bundled into one hospitalization episode. Variables used in the episode creating process included claims identification key, patient identification key, insurance type, main diagnosis code, treatment type, treatment start date, and treatment end date.

### 2.4. Main Descriptive Variables

The main descriptive variables were frequency and cost of medical care without addressing a specific hypothesis. Frequency included the number of hospitalizations and outpatient visits in Western medicine and traditional Korean medicine clinics, intervention (surgical and nonsurgical), and annual usage. Rehabilitation-related nonsurgical interventions were classified according to National Evidence-based Healthcare Collaborating Agency reports [20].

Cost included average cost per patient and cost per day (per diem) for joint disorders. Medical costs determined to be eligible for reimbursement by HIRA out of treatment costs were indicated in the submitted insurance claim statement. Medical costs, that is, the sum of benefits reimbursed by the insurer (Korean National Health Insurance Service) to the medical care institutions, were classified as INSUP and self-payment costs paid by the beneficiary (patient) as SLF. It was expressed as total treatment cost in Korean Won (1,000,000 KRW). Each patient's medical costs were calculated as the sum of costs listed on their claims. The average treatment amount was the amount of total medical expenses for one year divided by the number of patients. Per diem was the amount of total medical expenses for one year divided by the number of days of hospitalization or in an outpatient clinic.

The number of reimbursed days included the number of hospitalized days or outpatient visits and in-care drug prescription days. These days were defined based on the number of visits (for outpatient departments) or the length of hospital stay (for inpatient departments) of patients indicated in the submitted insurance claim statement [14]. Days per episode were calculated as total reimbursed days divided by the total number of episodes. Patient and medical institution-related characteristics are defined as follows.

### 2.5. Patient Characteristics

Patient characteristics included gender, age, medical insurance type, severity of disease, existence of surgery, and type of medicine. The main attending hospital characteristics included hospital type, region, ownership, the number of beds, the number of Western doctors, and the number of traditional medical practitioners. Patient demographic data obtained from the NHI claims database included gender, age, and medical insurance type (NHI, Medicaid, and others) at the date of visit of a health care institution and the most frequently visited ones. Individuals were qualified for Medicaid if they had a household income of less than $600 per month. Medical services for veterans and beneficiaries were free of charge as government expenditure. Severity was measured using the Charlson Comorbidity Index (CCI) [21] defined as the sum of weights related to each condition for which a patient had available claim data. The CCI score was determined based on the presence of specific ICD-10 codes during one year [22]. In this study, CCI at initiation was used as the CCI score of each patient.

### 2.6. Medical Institution Characteristics

The types of medical practice were divided into three as follows: traditional, Western, and both traditional and Western. Hospital was the main attending medical institution which was visited most frequently by the patient for care. If visit frequency per institution was the same, the main attending hospital was the last health care institution that the patient visited. Medical institutions included Tertiary and General Hospitals, hospitals, long-term care hospitals, Western Clinics, Dental Hospitals and Clinics, Public Health Hospitals (admission facility-equipped health center), Public Health Centers, Local Public Health Clinics, Traditional Hospitals, and Traditional Clinics. Region and ownership were the characteristics of the medical institution that the patient visited.

### 2.7. Statistical Analysis

Basic characteristics of the study sample are presented as frequencies and percentages. They are presented for each operational definition. Descriptive statistics are presented as type of care and hospital type. All statistical analyses were performed using SPSS version 21 for Windows (IBM Corp., Armonk, NY, USA).

## 3. Results

The general characteristics of the study population are summarized in Table 1.

A total of 1,100,018 patients were included, including 456,642 (41.5%) males and 643,376 (58.5%) females. All four years (from 2011 to 2014) showed higher percentages of females than males. Patients under 29 years of age accounted for the largest proportion (23.3%), followed by patients in their 50s (20.5%) and 60s (16.8%). A total of 1,050,691 (95.5%) patients were enrolled in the NHI scheme while the remaining 49,012 patients (4.5%) were enrolled in Medicaid. Patients with knee arthrodesis accounted for the most (24.2%), followed by those with foot joint disease. More than half (52.5%) of these patients had knee arthropathy. Approximately 70% (70.5%) of patients had mild joint disorder with CCI score of 0. Among the 1,100,018 patients, 18,041 (1.6%) patients underwent surgery while the majority (98.4%) of patients underwent nonsurgical procedures. For body regions where basic physical therapy was performed more than three times, the knee and shoulder regions accounted for more than 25%. For regions that needed surgery, the knees accounted for the most. For regions that underwent acupuncture two times or more, the shoulder, hand, and foot areas accounted for 30% or more (Table 2). Regardless of disease type, only 1.6% of patients underwent surgery, of which knee surgery was the most frequently performed type (43%~44%). The results of nonsurgical intervention distribution are shown in Table 3. The main attending medical institutions included 58,245 (5.3%) Tertiary and General Hospitals, 118,408 (10.8%) hospitals, 8,638 (0.8%) Western Clinics, 592,155 (53.8%) long-term care hospitals, 6,473 (29.6%) Traditional Hospitals, and (28.7%) Traditional Clinics. However, the results were different for hospitalization and outpatient visits (Table 4). Hospitalization was mainly in the order of hospitals > long-term care hospital > Western Clinic. This is mainly due to the characteristics of patients who require surgery. On the other hand, among the same primary clinic institutions, Traditional Clinics showed a higher proportion than Western Clinic for outpatients. The admission rates were 96.90% in Western medicine clinic and 3.10% in traditional Korean medicine clinics. Among all outpatient visits, 67.85% involved orthodox medicine while 32.15% involved traditional Korean medicine. In Western medicine clinics, patients were hospitalized most frequently in hospitals, followed by long-term care hospitals. Hospitalization at hospital level gradually decreased from 37.17% in 2011 to 36.37% in 2014. On the other hand, the percentage of patients who were hospitalized mainly in Traditional Hospitals increased from 2.12% in 2011 to 3.28% in 2014. Outpatient visits accounted for most visits to hospitals (Western medicine: 56.26%; Traditional Korean medicine: 31.69%). While the percentage of outpatients at Western medicine clinics steadily increased from 66.19% in 2011 to 68.93% by 2014, the percentage of outpatients at traditional Korean medicine clinics steadily decreased from 33.81% in 2011 to 31.07% in 2014. Most (91.0%) institutions were privately owned, and most (80.4%) of them were located outside Seoul. The majority (95.2%) of institutions had fewer than 5 beds. There was no significant difference among the four groups according to year. The total average treatment cost (RPE) is the sum of INSUP and SLF paid to medical care institution. RPE per patient was 185,933 KRW in 2011, 192,290 KRW in 2012, 202,967 KRW in 2013, and 208,739 KRW in 2014. Women incurred more medical expenses in 2011 to 2014 compared to men. Expenditure was increased with age. It peaked in patients in their 70s with a minimum of 377,448 KRW to a maximum of 388,445 KRW. In terms of expense by the type of joint lesion, knee lesions (M17) had the highest expense among the four types of joint disorders, followed by shoulder lesions (M75). As the severity of lesion was increased, the expense was also increased. However, the difference in expense was not statistically insignificant. Patients who were hospitalized spent 20 times more than those who were not hospitalized. The average treatment costs per patient in inpatient care and outpatient care were 192,414 and 65,319 KRW, respectively. Patients who used only traditional Korean medicine spent twice less than those who only used Western medicine. The range of RPE for Western medicine was from 181,225 KRW to 198,661 KRW. The range of RPE for traditional Korean medicine was from 82,019 KRW to 96,325 KRW. There were no differences in costs over 400,000 KRW among hospitals that practiced Western medicine (Tertiary and General Hospital, hospital, and Western Clinic). Costs for Western medicine hospitals were the highest, followed by that for Traditional Hospitals and long-term care hospitals (Table 5).

The frequency and total medical expenditures for Western medicine and traditional Korean medicine are shown in Table 6. There were 21,894,252 claims with a cost of 168,024,474 (1000 KRW) for Western medicine. However, there were only 9,628,946 claims with a cost of 38,602,696 (1000 KRW) for traditional Korean medicine. The medical expense per visit in an outpatient clinic was 22,000 KRW for Western medicine and about 18,000 KRW for traditional Korean medicine. The day per episode of traditional Korean medicine was longer than that of Western medicine. After analyzing the medical cost of claims for Western medicine and traditional Korean medicine, the proportion of each item was different. For Western medicine, the proportion of psychiatric costs was the highest (28.78%), followed by doctors' fees (27.7%), injections (16.59%), radiotherapy costs (8.74%), and laboratory costs (7.09%). For traditional Korean medicine, the proportion of doctors' fees was the highest (26.48%), followed by procedural costs (25.16%), injections (13.52%), admission costs (9.23%), and psychiatric costs (7.26%). Regarding traditional Korean medicine, most (70%) medical treatment costs were procedural costs and treatment costs. Doctors' fees accounted for only 21.54% of the total cost, similar to doctors' fees for Western medicine. Procedural costs accounted for the most (56.45%) among total cost for Western medicine. The second largest proportion was doctors' fees (40.49%). Admission costs, medication costs, and laboratory costs comprised less than 1% (Table 7).

In Table 8, it was not possible to use the inspection and image capturing system of 0 only in the Traditional Clinic because of legal restrictions. According to the region of disease, the knee accounted for the most, followed by the shoulder, foot, and hand in terms of hospitalization and outpatient visits. As the years progressed, the number of inpatient and outpatient visits was also increased for all body regions. Among the hospitalized patients, the number of claims for all years after 2011 increased the most for shoulder joints (78.43%) compared to 2011, followed by knees (61.93%), foot (50.72%), and hands (16.29%). On the other hand, outpatient cases occurred in the following order based on the location of the disease: hand (11.24%) > knee (8.49%) > ankle (9.63%) > shoulder (4.35%). According to time (year), difference in current usage patterns was especially different between Western medicine and traditional Korean medicine. Particularly, hospitalization increases for the knee and shoulder areas (shoulder: 105.00%, knee: 250.00%) in traditional Korean medicine were higher than those in Western medicine (shoulder: 77.99%, knee: 58.04%). The proportion of outpatient visits for the hand region in traditional Korean medicine increased steadily (2012: 4.31%, 2013: 14.49%, 2014: 13.46%). However, the shoulder area showed steady decrease (2011: −8.31%, 2012: −9.73%, 2014: −16.45%) (Table 9).

The costs and length of hospitalization by year are shown in Tables 10–17. Basic physical therapy was the most common nonsurgical intervention in Western medicine while acupuncture was the most common nonsurgical intervention in traditional Korean medicine. Both procedures are steady treatments that require two or more treatments. The proportions of acupuncture and basic physical therapy are almost equal (Table 18).

## 4. Discussion

This study assessed the prevalence and costs of most frequently used treatments for joint disorders in Korea to provide basic information for future usual care guidelines that may reduce health expenditures and help solve National Health Insurance deficits. This study used the 2011–2014 HIRA-NPS data consisting of 3% age-stratified and gender-stratified random samples. It appropriately reflected the South Korean population of 2011–2014 to capture real-world medical use and cost in joint disorders.

The results of the study showed that the proportion of female patients was higher compared to that of male patients. This is consistent with previous findings showing that women are more likely to utilize health care than men [23, 24]. This might be due to gender role differences such as occupation, hours of work, and occupational activities including housework and biological factors. Women are typically responsible for childcare and housework while men are typically expected to have a job [25].

The shoulder and knee joints accounted for the most hospital visits and increased steeply. In Korea, musculoskeletal disease accounted for 28.2% of National Health Insurance Corporation (NHIC) inpatient and outpatient claims. Knee joint disease has been ranked the 6th among reasons for inpatient care visit and the 5th among reasons for outpatient care visit among the population aged 65 years or older [26]. The incidence of gonarthrosis has been steadily increasing in Korea. Its rate in women was much higher than that in men [27].

While Western Clinics were the most frequently visited medical institution type between 2011 and 2014, the finding that Traditional Clinics were the next most frequently visited in this study was noteworthy. The Korean medical system is characterized by both Western and traditional Korean medical practices. In 2014, the number of claims from Western medicine was 49,031 for Tertiary and General Hospitals and 16,935 for hospitals and clinics. On the contrary, the number of claims from traditional Korean medicine was 14,729 for hospitals and 7,690 for clinics [26]. These circumstances reflect the high proportion of traditional Korean medicine use for joint disorders [9]. Our results are consistent with previous results showing that Traditional Clinics are the second most visited institution by patients with nonspecific low back pain [14].

Despite the high demand for traditional Korean medicine for musculoskeletal diseases, traditional Korean medical practitioners are precluded from diagnosing joint disorders independently due to regulatory restrictions in imaging device use. We confirmed this fact again by comparing subgroup costs related to the type of medicine in total treatment cost. Apart from procedures such as acupuncture, moxibustion, and cupping, many treatments were not covered by the NHI (Table 7). Large variations in diagnostic and therapeutic management between Western medicine and traditional Korean medicine indicate that more items in Korean medicine need to be covered and developed. Among the hospitalized patients, the number of claims for all years after 2011 increased mostly for shoulder joints (78.43%) compared to 2011, followed by knees (61.93%), foot (50.72%), and hands (16.29%). While the rate of use of traditional Korean medicine for the shoulder region slightly decreased in outpatient care, the number of hospitalizations increased sharply (Table 9). It is interesting that the proportion of traditional hospitalization increase for the knee and shoulder regions (shoulder: 105.00%, knee: 250.00%) was higher than that of Western medicine clinic hospitalization (shoulder: 77.99%, knee: 58.04%). In Korea, the medical delivery system of traditional medicine is not strict. Individuals can choose to visit primary medical institutions (Traditional Clinic) and higher medical institutions (Traditional Hospital). Shoulder and knee joint diseases are common musculoskeletal diseases. They are usually treated in primary care settings. However, if there is no response or a lack of effectiveness in primary care, a Traditional Hospital is attended for a more accurate diagnosis and evaluation. This might be the reason why there is increase in hospitalization in Traditional Hospitals that hire orthodox medical practitioners who can use X-rays and magnetic resonance imaging. Besides, because traditional medical practitioners cannot use these examination devices in a Traditional Clinic, outpatient care in Traditional Clinics was much lower compared to that in Western Clinics. Although simple radiology is helpful in the diagnosis of joint disease [28], patient might suffer the inconvenience of going to both Western and Traditional Clinics for accurate diagnosis due to legal restrictions [29].

Traditional Korean medicine had lower medical expenditures than Western medicine (inpatient care cost: Western medicine clinic, 160,000 KRW; traditional Korean medicine clinic, 50,000 KRW; outpatient care cost: Western medicine, 22,000 KRW; traditional Korean medicine, 18,000 KRW). The average treatment cost for traditional Korean medicine was lower compared to that for Western medicine. RPE ranged from 181,225 KRW to 198,661 KRW for Western medicine and 82,019 KRW to 96,325 KRW for traditional Korean medicine. In addition, out-of-pocket expenses for Western medicine (44,240 KRW to 49,621 KRW) were higher than those of traditional Korean medicine (20,154 KRW to 22966 KRW). Although expenditures for traditional Korean medicine were significantly lower compared to those for Western medicine, daily cost amount showed no significant difference between the two depending on the year. These results are similar to those of a previous study [6].

Most patients had mild joint diseases (more than 70%) with CCI scores of 0 and underwent nonsurgical treatment. Regardless of disease type, the proportion of surgery was less than 1%. Therefore, traditional medical care can serve as an alternative to Western medical care. We found that the proportion of acupuncture was slightly higher than basic physical therapy (Table 18). However, further research is needed to confirm that traditional Korean medicine is cost-effective for managing joint diseases.

This study has several limitations. First, the study was descriptive in nature. It reported sociodemographic characteristics, procedures, medication, and average cost for treating joint disorders without addressing a specific hypothesis. Recently, research results have been utilized as basis for policies by utilizing health-related big data. However, there is a lack of data analyzing various patterns of traditional medical services [30]. Although there are studies that use NHI claims data, they are limited to a single year or disease range [8, 13]. This study is novel in that it compared the utilization of Western medicine and traditional Korean medicine for the treatment of joint disorders in Korea. We believe the current study would serve as a good reference for countries with similar medical systems as that of Korea and would be able to contribute to international literature. Further research is required, such as analysis of factors influencing the use or frequency of Korean traditional medicine using multivariate statistics.

Second, while fee-for-service for nationally covered health care service was comprehensively recorded in the claim database, nonreimbursable items such as traditional drugs did not generate billing data. In addition, we only calculated direct medical costs based on information in the claim database. In general, there are nonmedical costs such as transportation costs and lost productivity due to morbidity because joint diseases tend to be chronic. In addition, the costs did not uncover items based on claims data that only contained information about medical services provided under the NHI. If uncovered items were included, the costs for traditional Korean medicine might be higher than that of Western medicine. In a previous study [31] comparing Western and traditional Korean medicine users, it was found that the traditional Korean medicine user group paid significantly more medical expenses than the Western medicine user group. In a study on the determinants of traditional Korean medicine use based on panel data [32], the number of patients using traditional Korean medicine was significantly higher than those using Western medicine. This is because the insurance benefit for traditional Korean medicine is lower than that for Western medicine.

Third, we did not include essential factors influencing choice of medical practice, such as education level, income, residence, severity, and health-related risk factors (e.g., alcohol consumption, smoking, and exercise) [33]. Previous studies have shown that factors affecting the use of traditional Korean medicine are not related to education level or income level [34, 35], high education level [14], low education level [36, 37], and low or high-income level [32]. In previous studies, factors such as the use of a therapist [33], confidence in oriental medical institution [32], recognition of therapeutic effect [38], and coping attitude of the oriental medical treatment [39] have been found to be significant factors. Although these essential variables are important parameters in choosing hospitals, they were missed due to the nature of the claims data. To overcome the omission of disturbance variables, medical insurance type (NHI or Medicaid) and region of hospital were used as surrogates of income and residence in this study. In addition, we considered disease severity by using CCI because severity of disease might greatly affect hospital choice. The remaining factors have been judged due to their impacts on health care utilization. We believe that the direction of the analysis of this study will not change. Due to the limitation in data characteristics, these elements were not included in this study. Future studies considering these factors are needed to confirm our findings.

Finally, the accuracy of diagnoses has been an issue due to the nature of claims data collected with the purpose of reimbursing health care services and not for clinical purposes [18]. The accuracy of diagnosis in the KNHI claims data has been reported to be about 70% [40]. Moreover, the accuracy of disease classification has been reported to be higher for inpatients than for outpatients. It is higher for severe disorders than for common mild disorders. It is also higher in General Hospitals than in clinics [41]. Nonetheless, in the process of designing this study, physicians in current practice concurred that these codes were not clearly differentiated for diagnosis in actual clinical practice settings in Korea. Therefore, analysis was performed in primary and secondary diagnoses in accordance with the opinion that various issues should be taken into account (e.g., private insurance, medical care institution characteristics, and individual differences in physicians) in category division. In addition, primary and secondary diagnoses are generally used in conjunction [14]. Therefore, in defining medical care usage due to joint disease, we reviewed not only the major diagnosis code, but also secondary diagnosis code. Despite our efforts, the diagnosis accuracy for joint diseases in this study might be challenged.

Despite these limitations, our study has several strengths. First, we analyzed age-stratified and gender-stratified random samples of the KNHI claims database representing 98% of the South Korean population. Claims statements covered extensive information on health care interventions (e.g., treatment, procedures, diagnostic tests, and prescription drugs), diagnosis, NHI payment cost, beneficiaries' out-of-pocket expenses, sociodemographic characteristics, and medical institutions, thus providing useful nationwide epidemiological data. Its representativeness, reliability, and validity have been confirmed previously [41]. However, there is a lack of data necessary to understand various consumption patterns and supply patterns of traditional medical services [30].

Second, there are studies in other countries that analyze the status of traditional Korean medicine utilization by using representative data source [30, 42–44]. Unlike most previous clinical studies whose duration was less than one year, we attempted to analyze the change over four years for joint diseases based on the type of medicine used (Western or traditional Korean medicine). Until now, no studies have reported national data on the management of joint disorders for 2011 to 2014. This study holds significance in that it is the first study that reports distinct differences in patterns of medical care use and costs between Western medicine and traditional Korean medicine.

An added strength of this study was that it provided patterns of complementary and alternative medicine treatment for joint disorders in Korea by covering traditional Korean medicine treatments as acupuncture, moxibustion, and cupping in the NHI.

Third, we constructed pilot medical episode data considering characteristics of health claim data for joint diseases. This can be used as a data processing technique to calculate basic dynamics information. Health insurance claim data were produced by physicians based on diagnosis at the first visit to the hospital. Related claim data were then produced, including the diagnosis code, the date of initiation of treatment (hospitalization or outpatient), and personal information (age, sex). To use epidemiological data, it is necessary to link the billing statement classified for administrative purposes to the same hospitalization case [45]. In this study, the same patient filed a billing statement with the same hospital in the same medical institution for inpatient care. The date was connected to one hospitalization case.

Our study has several policy implications. As disease structure can change from acute to chronic degenerative disease, interest in traditional medicine is increasing with aging. The main purpose of traditional medicine use in South Korea is to prevent disease and promote general health [10]. Though traditional medicine plays a substantial role in the Korean health care system, the annual number of health insurance claims from traditional Korean medicine institutions has stagnated and decreased since 2012 [46]. Medical use is affected by demographic, socioeconomic, and psychocultural factors. It has been reported that these factors can affect health care utilization by interactions between factors rather than independent factors [47, 48]. Therefore, it is important to grasp the current position of traditional Korean medicine in order to prepare policy and directives. Currently, difference in standards of practice underlies mistrust for traditional Korean medicine among Western medical practitioners [6]. To overcome conflicts among orthodox and traditional practitioners, we need an effective health care delivery system that encourages consultation for both Western and traditional Korean medicine with accessibility. Further discussion must be considered by providing consultation programs for other chronic diseases and joint diseases.

## 5. Conclusions

This study provided objective information about epidemiologic characteristics of patients with joint disorders treated with Western medicine and traditional Korean medicine. It provided an understanding about the recent status and trends. It will provide a basis for further expansion of traditional Korean medicine for patients with muscular disorders. Based on HIRA data, medical use for joint disorders showed significant difference between the groups. It provides basic information for future usual care guidelines linked to health policy and budget appropriation. Timely and accurate information is essential for policy-makers to make decisions. The results of our study will contribute to management decisions for musculoskeletal diseases involving joint disorders.

## Figures and Tables

**Table 1:** General characteristics of patients and hospitals.

Category	2011		2012		2013		2014		Total	
	*N*	(%)	*N*	(%)	*N*	(%)	*N*	(%)	*N*	(%)
Patient total	268,048	(100)	273,752	(100)	278,170	(100)	280,048	(100)	1,100,018	(100.0)
Gender										
Male	111,101	(41.4)	113,592	(41.5)	115,219	(41.4)	116,730	(41.7)	456,642	(41.5)
Female	156,947	(58.6)	160,160	(58.5)	162,951	(58.6)	163,318	(58.3)	643,376	(58.5)
Age (yr)										
≦29	64,226	(24.0)	64,564	(23.6)	63,712	(22.9)	63,696	(22.7)	256,198	(23.3)
30–39	27,303	(10.2)	27,272	(10.0)	27,047	(9.7)	27,176	(9.7)	108,798	(9.9)
40–49	42,286	(15.8)	41,452	(15.1)	41,118	(14.8)	40,898	(14.6)	165,754	(15.1)
50–59	53,565	(20.0)	56,005	(20.5)	58,252	(20.9)	58,201	(20.8)	226,023	(20.5)
60–69	44,704	(16.7)	46,016	(16.8)	46,949	(16.9)	47,162	(16.8)	184,831	(16.8)
70–79	35,964	(13.4)	38,443	(14.0)	41,092	(14.8)	42,915	(15.3)	158,414	(14.4)
Medical insurance type^*∗*^										
National Health Insurance	255,160	(95.2)	261,381	(95.5)	266,015	(95.6)	268,135	(95.7)	1,050,691	(95.5)
Medicaid	12,828	(4.8)	12,298	(4.5)	12,069	(4.3)	11,817	(4.2)	49,012	(4.5)
Others	60	(.0)	73	(.0)	86	(.0)	96	(.0)	315	(.0)
Lesion of joint^†^										
M17	141,148	(52.6)	143,697	(52.4)	145,346	(52.3)	147,274	(52.6)	577,465	(52.5)
M75^&^	16,677	(6.2)	16,401	(6.0)	17,223	(6.2)	16,858	(6.0)	67,159	(6.1)
S63	46,659	(17.4)	47,900	(17.5)	48,776	(17.6)	49,269	(17.6)	192,604	(17.5)
S93	63,564	(23.7)	65,754	(24.0)	66,825	(24.0)	66,647	(23.8)	262,790	(23.9)
Severity (CCI)^‡^										
0	157,575	(74.4)	154,798	(74.4)	151,540	(74.3)	115,851	(58.4)	579,764	(70.5)
1	37,600	(17.7)	37,236	(17.9)	36,331	(17.8)	49,180	(24.8)	160,347	(19.5)
2	11,630	(5.5)	11,245	(5.4)	11,097	(5.4)	21,684	(10.9)	55,656	(6.8)
3+	5,041	(2.4)	4,792	(2.3)	4,874	(2.4)	11,556	(5.8)	26,263	(3.2)
Surgery										
No	264,211	(98.6)	269,325	(98.4)	273,412	(98.3)	275,029	(98.2)	1,081,977	(98.4)
Yes	3,837	(1.4)	4,427	(1.6)	4,758	(1.7)	5,019	(1.8)	18,041	(1.6)
Patient total	268,048	(100.0)	273,752	(100.0)	278,170	(100.0)	280,048	(100.0)	1,100,018	(100.0)
Type of medicine										
Traditional	64,453	(24.1)	63,426	(23.2)	63,234	(22.7)	61,159	(21.8)	252,272	(22.9)
Both	40,901	(15.3)	40,824	(14.9)	41,482	(14.9)	40,793	(14.6)	164,000	(14.9)
Western	162,550	(60.7)	169,502	(61.9)	173,454	(62.4)	178,096	(63.6)	683,602	(62.2)
Hospital type^§^										
Tertiary and General Hospital	13,100	(4.9)	14,125	(5.2)	14,911	(5.4)	16,109	(5.8)	58,245	(5.3)
Hospital	25,672	(9.6)	29,657	(10.8)	30,968	(11.1)	32,111	(11.5)	118,408	(10.8)
Western Clinic	1,912	(.7)	2,138	(.8)	2,268	(.8)	2,320	(.8)	8,638	(.8)
Long-term care hospital	145,278	(54.2)	146,694	(53.6)	149,037	(53.6)	151,146	(54.0)	592,155	(53.8)
Traditional Hospital	1,631	(.6)	1,631	(.6)	1,628	(.6)	1,583	(.6)	6,473	(.6)
Traditional Clinic	80,423	(30.0)	79,507	(29.0)	79,357	(28.5)	76,779	(27.4)	316,066	(28.7)
Region										
Seoul (Urban)	52,548	(19.6)	53,682	(19.6)	54,538	(19.6)	54,907	(19.6)	215,675	(19.6)
Metropolitan city	68,101	(25.4)	70,626	(25.8)	72,117	(25.9)	72,436	(25.9)	283,280	(25.8)
Other (Rural)	147,399	(55.0)	149,444	(54.6)	151,515	(54.5)	152,705	(54.5)	601,063	(54.6)
Ownership										
Public	1,894	(.7)	1,916	(.7)	2,018	(.7)	2,120	(.8)	7,948	(.7)
Corporation	20,395	(7.6)	22,750	(8.3)	23,386	(8.4)	24,272	(8.7)	90,803	(8.3)
Private	245,726	(91.7)	249,084	(91.0)	252,765	(90.9)	253,654	(90.6)	1,001,229	(91.0)
Number of beds										
≦5	254,565	(95.0)	261,339	(95.5)	265,257	(95.4)	266,216	(95.1)	1,047,377	(95.2)
6–12	8,455	(3.2)	7,626	(2.8)	7,959	(2.9)	8,635	(3.1)	32,675	(3.0)
13–17	2,195	(.8)	2,444	(.9)	2,401	(.9)	2,542	(.9)	9,582	(.9)
18≦	2,820	(1.1)	2,343	(.9)	2,552	(.9)	2,655	(.9)	10,370	(.9)
Number of Western doctors per 100 beds										
≦4	172,528	(64.4)	257,710	(94.3)	260,076	(93.7)	260,036	(93.0)	950,350	(86.5)
5–11	70,428	(26.3)	11,177	(4.1)	12,686	(4.6)	14,624	(5.2)	108,915	(9.9)
12–19	13,829	(5.2)	2,055	(.8)	1,897	(.7)	2,451	(.9)	20,232	(1.8)
20≦	11,231	(4.2)	2,469	(.9)	2,976	(1.1)	2,474	(.9)	19,150	(1.7)
Number of traditional doctors per 100 beds										
0	262,580	(98.0)	270,211	(99.3)	274,766	(99.4)	276,741	(99.4)	1,084,298	(99.0)
1≦	5,436	(2.0)	1,796	(.7)	1,753	(.6)	1,686	(.6)	10,671	(1.0)

^*∗*^Medical insurance type divided into National Health Insurance, Medicaid, and others. Others include veterans and beneficiaries who receive care free of charge as a government expenditure. ^†^When one patient had multiple joint diseases, the most frequent disease is indicated. ^&^A case involving one or more of the other three joint disorders which were included in this study. ^‡^Severity was measured using the Charlson Comorbidity Index (CCI), defined as the sum of weights related to each condition for which a patient submitted claims. ^§^Hospital type is the type of medical institution most frequently visited.

**Table 2:** Distribution of nonsurgical intervention in both WM and traditional medicine according to the corporal name.

Nonsurgical intervention			2011					2012				
			Total	M17	M75	S63	S93	Total	M17	M75	S63	S93
		Total (*N*^*∗*^)	1,481,969	648,757	447,224	136,972	249,016	1,560,032	683,247	471,040	144,044	261,701

WM	Basic physical therapy^†^	0										
		*N*	947,982	377,024	293,443	101,240	176,275	1,000,863	410,595	298,619	106,230	185,419
		%	(63.97)	(58.11)	(65.61)	(73.91)	(70.79)	(64.16)	(60.09)	(63.40)	(73.75)	(70.85)
		1										
		*N*	32,582	13,360	6,568	6,504	6,150	38,311	14,819	9,081	7,424	6,987
		%	(2.20)	(2.06)	(1.47)	(4.75)	(2.47)	(2.46)	(2.17)	(1.93)	(5.15)	(2.67)
		2										
		*N*	136,773	77,169	33,618	7,892	18,094	137,871	72,622	38,010	8,468	18,771
		%	(9.23)	(11.89)	(7.52)	(5.76)	(7.27)	(8.84)	(10.63)	(8.07)	(5.88)	(7.17)
		3≦										
		*N*	364,632	181,204	113,595	21,336	48,497	382,987	185,211	125,330	21,922	50,524
		%	(24.60)	(27.93)	(25.40)	(15.58)	(19.48)	(24.55)	(27.11)	(26.61)	(15.22)	(19.31)

WM	Simple rehabilitation^‡^	0										
		*N*	1,475,271	647,748	446,473	132,184	248,866	1,551,724	681,736	469,564	139,023	261,401
		%	(99.55)	(99.84)	(99.83)	(96.50)	(99.94)	(99.47)	(99.78)	(99.69)	(96.51)	(99.89)
		1≦										
		*N*	6,698	1,009	751	4,788	150	8,308	1,511	1,476	5,021	300
		%	(.45)	(.16)	(.17)	(3.50)	(.06)	(.53)	(.22)	(.31)	(3.49)	(.11)

WM	Professional rehabilitation^§^	0										
		*N*	1,481,873	648,725	447,185	136,949	249,014	1,560,008	683,235	471,031	144,041	261,701
		%	(99.99)	(100)	(99.99)	(99.98)	(100)	(100)	(100)	(100)	(100)	(100)
		1≦										
		*N*	96	32	39	23	2	24	12	9	3	—
		%	(.01)	(.00)	(.01)	(.02)	(.00)	(.00)	(.00)	(.00)	(.00)	(.00)

WM	Rehabilitation of CNS	0										
		*N*	1,481,962	648,751	447,223	136,972	249,016	1,560,029	683,244	471,040	144,044	261,701
		%	(100)	(100)	(100)	(100)	(100)	(100)	(100)	(100)	(100)	(100)
		1≦										
		*N*	7	6	1	—	—	3	3	—	—	—
		%	(.00)	(.00)	(.00)	(.00)	(.00)	(.00)	(.00)	(.00)	(.00)	(.00)

TM	Acupuncture	0										
		*N*	982,471	512,929	261,161	84,032	124,349	1,062,109	540,051	299,859	88,627	133,572
		%	(66.29)	(79.06)	(58.40)	(61.35)	(49.94)	(68.08)	(79.04)	(63.66)	(61.53)	(51.04)
		1										
		*N*	40,678	12,784	14,083	4,402	9,409	44,984	15,421	14,765	4,728	10,070
		%	(2.74)	(1.97)	(3.15)	(3.21)	(3.78)	(2.88)	(2.26)	(3.13)	(3.28)	(3.85)
		2≦										
		*N*	458,820	123,044	171,980	48,538	115,258	452,939	127,775	156,416	50,689	118,059
		%	(30.96)	(18.97)	(38.46)	(35.44)	(46.29)	(29.03)	(18.70)	(33.21)	(35.19)	(45.11)

TM	Moxibustion	0										
		*N*	1,481,922	648,728	447,214	136,969	249,011	1,559,985	683,210	471,032	144,043	261,700
		%	(100)	(100)	(100)	(100)	(100)	(100)	(100)	(100)	(100)	(100)
		1≦										
		*N*	47	29	10	3	5	47	37	8	1	1
		%	(.00)	(.00)	(.00)	(.00)	(.00)	(.00)	(.01)	(.00)	(.00)	(.00)

TM	Cupping	0										
		*N*	1,481,712	648,669	447,115	136,964	248,964	1,559,844	683,132	470,982	144,040	261,690
		%	(99.98)	(99.99)	(99.98)	(99.99)	(99.98)	(99.99)	(99.98)	(99.99)	(100)	(100)
		1≦										
		*N*	257	88	109	8	52	188	115	58	4	11
		%	(.02)	(.01)	(.02)	(.01)	(.02)	(.01)	(.02)	(.01)	(.00)	(.00)

TM	Heat & cold therapy	0										
		*N*	1,481,856	648,712	447,194	136,964	248,986	1,559,985	683,225	471,027	144,042	261,691
		%	(99.99)	(99.99)	(99.99)	(99.99)	(99.99)	(100)	(100)	(100)	(100)	(100)
		1≦										
		*N*	113	45	30	8	30	47	22	13	2	10
		%	(.01)	(.01)	(.01)	(.01)	(.01)	(.00)	(.00)	(.00)	(.00)	(.00)

Nonsurgical intervention			2013					2014				
			Total	M17	M75	S63	S93	Total	M17	M75	S63	S93
		Total (*N*^*∗*^)	1,594,949	698,540	475,765	150,621	270,023	1,600,774	706,617	468,443	152,359	273,355

WM	Basic physical therapy^†^	0										
		*N*	1,034,024	428,800	302,461	112,026	190,737	1,033,566	432,591	295,844	112,726	192,405
		%	(64.83)	(61.39)	(63.57)	(74.38)	(70.64)	(64.57)	(61.22)	(63.15)	(73.99)	(70.39)
		1										
		*N*	46,856	21,507	10,255	7,147	7,947	45,063	18,589	10,106	7,624	8,744
		%	(2.94)	(3.08)	(2.16)	(4.75)	(2.94)	(2.82)	(2.63)	(2.16)	(5.00)	(3.20)
		2										
		*N*	126,126	62,627	34,839	9,016	19,644	122,500	59,726	33,139	9,146	20,489
		%	(7.91)	(8.97)	(7.32)	(5.99)	(7.27)	(7.65)	(8.45)	(7.07)	(6.00)	(7.50)
		3≦										
		*N*	387,943	185,606	128,210	22,432	51,695	399,645	195,711	129,354	22,863	51,717
		%	(24.32)	(26.57)	(26.95)	(14.89)	(19.14)	(24.97)	(27.70)	(27.61)	(15.01)	(18.92)

WM	Simple rehabilitation^‡^	0										
		*N*	1,585,834	696,682	474,267	145,176	269,709	1,590,708	704,479	466,442	146,791	272,996
		%	(99.43)	(99.73)	(99.69)	(96.38)	(99.88)	(99.37)	(99.70)	(99.57)	(96.35)	(99.87)
		1≦										
		*N*	9,115	1,858	1,498	5,445	314	10,066	2,138	2,001	5,568	359
		%	(.57)	(.27)	(.31)	(3.62)	(.12)	(.63)	(.30)	(.43)	(3.65)	(.13)

WM	Professional rehabilitation^§^	0										
		*N*	1,594,914	698,521	475,749	150,621	270,023	1,600,736	706,585	468,439	152,358	273,354
		%	(100)	(100)	(100)	(100)	(100)	(100)	(100)	(100)	(100)	(100)
		1≦										
		*N*	35	19	16	—	—	38	32	4	1	1
		%	(.00)	(.00)	(.00)	(.00)	(.00)	(.00)	(.00)	(.00)	(.00)	(.00)

WM	Rehabilitation of CNS	0										
		*N*	1,594,942	698,538	475,760	150,621	270,023	1,600,773	706,616	468,443	152,359	273,355
		%	(100)	(100)	(100)	(100)	(100)	(100)	(100)	(100)	(100)	(100)
		1≦										
		*N*	7	2	5	—	—	1	1	—	—	—
		%	(.00)	(.00)	(.00)	(.00)	(.00)	(.00)	(.00)	(.00)	(.00)	(.00)

TM	Acupuncture	0										
		*N*	1,085,224	549,587	307,484	89,800	138,353	1,102,972	559,982	309,311	92,009	141,670
		%	(68.04)	(78.68)	(64.63)	(59.62)	(51.24)	(68.90)	(79.25)	(66.03)	(60.39)	(51.83)
		1										
		*N*	55,124	23,007	16,632	5,186	10,299	50,447	20,075	15,875	4,656	9,841
		%	(3.46)	(3.29)	(3.50)	(3.44)	(3.81)	(3.15)	(2.84)	(3.39)	(3.06)	(3.60)
		2≦										
		*N*	454,601	125,946	151,649	55,635	121,371	447,355	126,560	143,257	55,694	121,844
		%	(28.50)	(18.03)	(31.87)	(36.94)	(44.95)	(27.95)	(17.91)	(30.58)	(36.55)	(44.57)

TM	Moxibustion	0										
		*N*	1,594,876	698,484	475,756	150,619	270,017	1,600,667	706,522	468,436	152,359	273,350
		%	(100.00)	(99.99)	(100)	(100)	(100)	(99.99)	(99.99)	(100)	(100)	(100)
		1≦										
		*N*	73	56	9	2	6	107	95	7	—	5
		%	(.00)	(.01)	(.00)	(.00)	(.00)	(.01)	(.01)	(.00)	(.00)	(.00)

TM	Cupping	0										
		*N*	1,594,897	698,507	475,754	150,621	270,015	1,600,518	706,427	468,397	152,357	273,337
		%	(100)	(100)	(100)	(100)	(100)	(99.98)	(99.97)	(99.99)	(100)	(100)
		1≦										
		*N*	52	33	11	—	8	256	190	46	2	18
		%	(.00)	(.00)	(.00)	(.00)	(.00)	(.02)	(.03)	(.01)	(.00)	(.01)

TM	Heat & cold therapy	0										
		*N*	1,594,897	698,507	475,754	150,621	270,015	1,600,734	706,584	468,439	152,358	273,353
		%	(100)	(100)	(100)	(100)	(100)	(100)	(100)	(100)	(100)	(100)
		1≦										
		*N*	52	33	11	—	8	40	33	4	1	2
		%	(.00)	(.00)	(.00)	(.00)	(.00)	(.00)	(.00)	(.00)	(.00)	(.00)

^*∗*^A patient could be hospitalized more than once during the study period, which resulted in more than one claim per patient. Thus, the number of claims in the study was higher than the number of patients. ^†^Basic physical therapy included superficial heat therapy, cold therapy, deep heat therapy, UV irradiation, transcutaneous electrical nerve stimulation, massage therapy, and simple therapeutic exercise. ^‡^Simple rehabilitation included paraffin bath, hydrotherapy, intermittent traction therapy, electrical stimulation therapy, laser therapy, therapeutic exercise, motor point block, pneumatic compression, complex decongestive physical therapy, and iontophoresis. ^§^Professional rehabilitation included pool therapy, occupational therapy, activities of daily living training, neurogenic bladder training, functional electrical stimulation therapy, myofascial trigger point injection, rehabilitative social work, rehabilitative breathing therapy, rehabilitative functional training, and rehabilitative dysphagia therapy. M17, knee lesions; M75, shoulder lesions; S63, wrist and hand level lesions; S93, ankle and foot level lesions; WM, Western medicine; TM, Korean traditional medicine.

**Table 3:** Comparison of rate of surgery by diagnostic code.

Year			Total		Knee lesions [M17]		Shoulder lesions [M75]		Wrist and hand level [S63]		Ankle and foot level [S93]	
			*N* ^*∗*^	%	*N*	%	*N*	%	*N*	%	*N*	%
	Total	Unit	1,481,969	100.00	648,757	43.78	447,224	30.18	136,972	9.24	249,016	16.80

2011	No	*N*	1,477,896		646,826		445,926		136,436		248,708	
		%	(99.73)		(99.70)		(99.71)		(99.61)		(99.88)	
	Yes	*N*	4,073		1,931		1,298		536		308	
		%	(.27)		(.30)		(.29)		(.39)		(.12)	

	Total	Unit	1,560,032	100.00	683,247	43.80	471,040	30.19	144,044	9.23	261,701	16.78

2012	No	*N*	1,555,319		681,193		469,334		143,450		261,342	
		%	(99.70)		(99.70)		(99.64)		(99.59)		(99.86)	
	Yes	*N*	4,713		2,054		1,706		594		359	
		%	(.30)		(.30)		(.36)		(.41)		(.14)	

	Total	Unit	1,594,949	100.00	698,540	43.80	475,765	29.83	150,621	9.44	270,023	16.93

2013	No	*N*	1,589,924		696,372		473,895		150,072		269,585	
		%	(99.68)		(99.69)		(99.61)		(99.64)		(99.84)	
	Yes	*N*	5,025		2,168		1,870		549		438	
		%	(.32)		(.31)		(.39)		(.36)		(.16)	

	Total	Unit	1,600,774	100.00	706,617	44.14	468,443	29.26	152,359	9.52	273,355	17.08

2014	No	*N*	1,595,458		704,410		466,431		151,772		272,845	
		%	(99.67)		(99.69)		(99.57)		(99.61)		(99.81)	
	Yes	*N*	5,316		2,207		2,012		587		510	
		%	(.33)		(.31)		(.43)		(.39)		(.19)	

^*∗*^A patient could be hospitalized more than once during the study period, which resulted in more than one claim per patient. Thus, the number of claims in the study was higher than the number of patients.

**Table 4:** Number of hospitalizations and outpatient visits by type of medicine in 2011–2014.

Type of medicine	Hospital type	Unit^*∗*^	2011		2012		2013		2014		2011–2014	
			H	O	H	O	H	O	H	O	H	O
WM	Tertiary Hospital	*N*	355	7,860	348	10,697	383	11,051	455	10,817	1,541	40,425
		%	4.08	0.53	3.20	0.69	3.05	0.70	3.19	0.68	3.32	0.65
	General Hospital	*N*	1,123	29,569	1,263	46,307	1,543	47,052	1,677	52,028	5,606	174,956
		%	12.92	2.01	11.61	2.99	12.29	2.97	11.74	3.28	12.08	2.83
	Hospital	*N*	3,232	62,241	4,061	127,047	4,493	131,644	5,193	136,668	16,979	457,600
		%	37.17	4.23	37.32	8.20	35.79	8.32	36.37	8.62	36.58	7.39
	Long-term care hospital	*N*	2,491	3,778	3,504	8,461	4,237	8,142	4,925	8,247	15,157	28,628
		%	28.65	0.26	32.20	0.55	33.75	0.51	34.49	0.52	32.66	0.46
	Western Clinic	*N*	1,296	867,535	1,383	858,553	1,493	874,403	1,508	881,056	5,680	3,481,547
		%	14.91	58.92	12.71	55.45	11.89	55.29	10.56	55.56	12.24	56.26
	Dental Hospital	*N*	0	14	0	38	0	11	0	18	0	81
		%	0.00	0.00	0.00	0.00	0.00	0.00	0.00	0.00	0.00	0.00
	Public Health Center	*N*	0	1,845	0	2,159	0	2,441	0	2,865	0	9,310
		%	0.00	0.13	0.00	0.14	0.00	0.15	0.00	0.18	0.00	0.15
	Local Public Health Clinic	*N*	0	442	0	487	0	245	0	236	0	1,410
		%	0.00	0.03	0.00	0.03	0.00	0.02	0.00	0.01	0.00	0.02
	Public Health Hospital	*N*	2	1,253	1	1,151	3	946	1	1,060	7	4,410
		%	0.02	0.09	0.01	0.07	0.02	0.06	0.01	0.07	0.02	0.07
	*Total*	*N*	*8,499*	*974,537*	*10,560*	*1,054,900*	*12,152*	*1,075,935*	*13,759*	*1,092,995*	*44,970*	*4,198,367*
		%	*97.75 *	*66.19 *	*97.05 *	*68.13 *	*96.79 *	*68.03 *	*96.35 *	*68.93 *	*96.90 *	*67.85 *

TM	Traditional Hospital	*N*	184	4,035	286	7,932	366	7,680	469	8,818	1,305	28,465
		%	2.12	0.27	2.63	0.51	2.92	0.49	3.28	0.56	2.81	0.46
	Traditional Clinic	*N*	12	493,842	35	485,603	37	497,972	52	483,833	136	1,961,250
		%	0.14	33.54	0.32	31.36	0.29	31.49	0.36	30.51	0.29	31.69
	*Total*	*N*	*196*	*497,877*	*321*	*493,535*	*403*	*505,652*	*521*	*492,651*	*1,441*	*1,989,715*
		%	*2.25 *	*33.81 *	*2.95 *	*31.87 *	*3.21 *	*31.97 *	*3.65 *	*31.07 *	*3.10 *	*32.15 *

Total		*N*	8,695	1,472,414	10,881	1,548,435	12,555	1,581,587	14,280	1,585,646	46,411	6,188,082
		%	100.00	100.00	100.00	100.00	100.00	100.00	100.00	100.00	100.00	100.00

^*∗*^Patients with overlapping records were tallied as one patient (overlap was not allowed). H, hospitalization; O, outpatient; WM, Western medicine; TM, Korean traditional medicine.

**Table 5:** Average treatment amount and benefit per patient by year.

Category	2011			2012			2013			2014		
	RPE^*∗*^	INSUP^†^	SLF^‡^	RPE^*∗*^	INSUP^†^	SLF^‡^	RPE^*∗*^	INSUP^†^	SLF^‡^	RPE^*∗*^	INSUP^†^	SLF^‡^
Total	185,933	140,190	45,052	192,290	144,552	47,009	202,967	152,456	49,617	208,739	156,519	51,398
Gender												
Male	130,549	95,580	33,405	135,980	99,443	34,934	145,561	106,375	37,180	153,724	112,670	39,211
Female	225,139	171,769	53,297	232,227	176,546	55,573	243,557	185,039	58,411	248,061	187,859	60,108
Age (yr)												
≦29	72,576	50,798	21,766	75,827	53,013	22,798	80,484	56,216	24,252	86,426	60,587	25,825
30–39	84,267	58,835	25,372	89,666	62,667	26,939	92,840	64,659	27,997	98,049	68,492	29,482
40–49	121,156	86,493	34,503	127,488	91,277	36,107	134,378	96,345	37,875	141,772	101,542	40,027
50–59	193,314	139,945	52,833	196,220	141,450	54,582	207,876	150,072	57,232	216,450	157,025	59,170
60–69	309,243	236,773	70,029	311,086	237,807	70,698	321,981	245,078	73,736	322,749	244,490	75,304
70–79	377,448	305,038	71,346	382,637	306,720	74,275	391,056	313,167	76,574	388,445	309,676	77,258
Medical insurance type												
NHI	180,246	132,778	46,862	187,175	137,685	48,839	198,249	146,009	51,511	204,759	150,730	53,296
Medicaid	298,500	288,278	9,262	300,467	291,371	8,384	307,033	295,660	8,220	298,439	289,135	8,745
Others	302,890	0	0	281,712	720	0	193,558	0	0	285,501	0	0
Lesion of joint												
M17	318,775	247,050	70,286	321,522	247,790	72,013	334,876	258,239	74,930	335,457	257,336	76,387
M17^&^	214,173	161,693	51,469	228,679	172,504	55,286	246,375	185,404	59,464	250,494	188,780	60,578
M75	273,798	204,867	68,361	287,629	215,195	71,603	299,415	223,468	75,254	322,791	240,597	81,185
S63	59,589	41,837	17,681	60,432	42,435	17,970	62,989	44,297	18,650	67,402	47,452	19,899
S63^&^	145,409	103,545	41,771	154,690	110,097	44,542	162,736	116,024	46,700	173,306	123,653	49,639
S93	80,779	56,892	23,850	83,765	58,891	24,844	88,453	62,166	26,253	94,310	66,510	27,765
Severity (CCI)												
0	203,081	153,881	48,398	209,020	157,743	50,440	221,676	167,186	53,420	157,944	115,659	42,148
1	199,087	150,625	47,563	211,708	159,868	50,817	223,060	168,461	53,604	273,424	207,024	65,595
2	212,462	161,156	50,725	204,987	155,273	49,011	230,624	174,488	55,662	371,890	287,348	82,390
3+	204,016	154,551	48,973	247,400	188,624	57,910	244,925	186,833	57,180	470,263	368,719	93,844
Hospital admission												
No	120,367	88,290	31,661	121,813	89,057	32,334	126,287	92,267	33,586	131,932	96,429	35,035
Yes	3,192,333	2,519,960	659,078	2,972,861	2,334,061	625,995	2,932,509	2,294,974	620,271	2,689,568	2,097,376	579,924
Inpatient visit												
1≦	172,322	129,370	42,262	178,367	133,526	44,130	187,228	140,111	46,235	192,414	143,780	47,833
Outpatient visit												
1	61,372	44,201	16,944	58,572	42,469	15,878	61,113	44,077	16,623	65,319	47,296	17,866
2-3	167,073	123,186	43,449	156,721	114,800	41,323	165,529	121,119	43,911	172,643	125,955	46,092
4≦	511,061	393,692	115,248	536,884	410,668	124,195	562,200	430,102	129,674	572,730	437,335	132,812
Type of medicine												
Traditional	82,019	61,845	20,154	86,266	65,161	21,076	90,827	68,827	21,979	96,325	73,323	22,966
Both	368,924	280,760	87,644	382,499	290,307	91,415	410,128	310,688	97,825	421,279	318,877	101,784
Western	181,225	135,985	44,240	186,151	139,155	46,017	194,305	145,102	48,164	198,661	147,900	49,621
Hospital type												
Tertiary and General Hospital	489,902	358,403	120,547	499,923	365,372	124,248	521,851	379,551	129,039	513,518	373,917	129,108
Hospital	400,853	302,498	97,522	400,137	301,007	97,878	403,412	303,041	99,363	401,838	299,704	101,105
Western Clinic	439,283	346,145	92,816	490,846	388,042	102,269	549,396	431,928	117,009	601,268	473,599	125,127
Long-term care hospital	151,730	115,386	36,252	152,247	115,222	36,955	161,165	121,970	39,115	164,468	124,275	40,104
Traditional Hospital	170,319	116,816	53,366	190,266	132,261	57,678	206,204	143,719	62,073	254,941	178,466	76,299
Traditional Clinic	123,954	93,265	30,612	126,000	94,782	31,157	133,370	100,470	32,823	138,372	104,463	33,798
Region												
Seoul (Urban)	193,889	144,031	48,933	204,091	151,668	51,127	207,939	153,673	52,466	217,649	161,097	55,268
Metropolitan city	206,956	156,123	49,511	205,397	154,325	49,914	222,903	167,268	54,395	224,130	168,026	54,704
Other (Rural)	173,384	131,459	41,609	181,856	137,378	44,157	191,688	144,969	46,317	198,235	149,414	48,438
Ownership												
Private	356,865	272,144	78,988	502,172	389,016	108,319	458,967	355,555	96,233	446,919	343,020	100,664
Corporation	399,808	296,961	96,104	395,387	293,375	95,243	414,433	306,100	100,105	428,235	315,244	105,371
Public	166,882	126,175	40,558	171,357	129,080	42,132	181,359	136,620	44,574	185,744	139,770	45,822

^*∗*^The sum of INSUP and SLF paid to the medical care institution. The total costs of items determined to be eligible for reimbursement by the HIRA (Health Insurance Review and Assessment Service) out of the total treatment amount were indicated in the submitted insurance claim statement. ^†^INSUP: the cost reimbursed by the Korean National Health Insurance Service as the insurer. ^‡^SLF: the self-payment amount paid by the beneficiary. ^&^A case involving one or more of the other three joint disorders which were included in this study.

**Table 6:** Comparison of medical costs by type of medicine in 2011–2014.

Unit	Year	Type of medicine	Frequency		Insurance charge			LOS
			*N* ^*∗*^	%	Cost^†^	%	Per diem^‡^	Days per episode^§^
H	2011	WM	8,499	97.75	16,542	99.37	162,276	10.98
		TM	196	2.25	105	0.63	53,097	12.23
		Total	8,695	100	16,647	100	159,815	11.01
	2012	WM	10,560	97.05	18,063	99.03	157,605	9.67
		TM	321	2.95	178	0.97	57,256	12.18
		Total	10,881	100	18,240	100	154,644	9.75
	2013	WM	12,152	96.79	19,969	98.65	155,222	9.31
		TM	403	3.21	216	1.35	62,217	10.78
		Total	12,555	100	20,185	100	152,237	9.35
	2014	WM	13,759	96.35	20,077	98.65	154,829	8.51
		TM	521	3.65	276	1.35	60,435	10.72
		Total	14,280	100	20,353	100	151,385	8.59

O	2011	WM	974,537	66.19	23,827	71.81	22,030	1.2
		TM	497,877	33.81	9,354	28.19	18,573	1.04
		Total	1,472,414	100	33,181	100	20,861	1.15
	2012	WM	1,054,900	68.13	24,848	72.24	23,555	1.12
		TM	493,535	31.87	9,551	27.76	19,352	1.03
		Total	1,548,435	100	34,399	100	22,215	1.09
	2013	WM	1,075,935	68.03	26,203	72.24	24,354	1.11
		TM	505,652	31.97	10,071	27.76	19,917	1.03
		Total	1,581,587	100	36,274	100	22,935	1.08
	2014	WM	1,092,995	68.93	27,887	73.19	25,514	1.1
		TM	492,651	31.07	10,218	26.81	20,740	1.03
		Total	1,585,646	100	38,104	100	24,031	1.08

^*∗*^A patient could be hospitalized more than once during the study period, which resulted in more than one claim per patient. Thus, the number of claims in the study was higher than the number of patients. ^†^Costs determined to be eligible for reimbursement by the HIRA (Health Insurance Review and Assessment Service) out of the total treatment amount were indicated in the submitted insurance claim statement. Costs are expressed in Korean Won (1,000,000 KRW). ^‡^Per diem is the average daily cost of services covered by National Health Insurance. It is expressed in Korean Won. ^§^Days per episode are the total number of reimbursed days divided by the total number of episodes. The number of reimbursed days includes the number of hospitalized days or outpatient visits and in-care drug prescription days. H, hospitalization; O, outpatient; WM, Western medicine; TM, Korean traditional medicine.

**Table 7:** Comparison of subgroup costs by type of medicine (2011–2014).

Type	Unit	Doctors' fees	Admission costs	Medication costs	Injection costs	Anesthesia costs	Physiotherapy costs	Psychotherapy costs	Procedural costs	Laboratory costs	Radiotherapy costs	None	Total^*∗*^
WM	*N*	6,064,234	178,833	614,097	3,632,780	697,677	6,300,815	194	791,129	1,551,682	1,914,274	148,537	21,894,252
	%	27.7	0.82	2.8	16.59	3.19	28.78	0	3.61	7.09	8.74	0.68	100
	KRW	44,499,914	15,515,383	2,477,317	22,718,121	9,271,746	12,202,897	3,897	42,278,059	5,393,884	10,074,191	3,589,065	168,024,474
	%	26.48	9.23	1.47	13.52	5.52	7.26	0	25.16	3.21	6	2.14	100

TM	*N*	2,074,061	6,648	332,810	0	0	0	3	6,932,215	8,192	0	275,017	9,628,946
	%	21.54	0.07	3.46	0	0	0	0	71.99	0.09	0	2.86	100
	KRW	15,631,220	592,114	208,776	0	0	0	31	21,791,571	31,038	0	347,946	38,602,696
	%	40.49	1.53	0.54	0	0	0	0	56.45	0.08	0	0.9	100

Total	*N*	8,138,295	185,481	946,907	3,632,780	697,677	6,300,815	197	7,723,344	1,559,874	1,914,274	423,554	31,523,198
	%	25.82	0.59	3	11.52	2.21	19.99	0	24.5	4.95	6.07	1.34	100
	KRW	60,131,134	16,107,497	2,686,093	22,718,121	9,271,746	12,202,897	3,929	64,069,631	5,424,922	10,074,191	3,937,011	206,627,170
	%	29.1	7.8	1.3	10.99	4.49	5.91	0	31.01	2.63	4.88	1.91	100

^*∗*^Costs determined to be eligible for reimbursement by the HIRA (Health Insurance Review and Assessment Service) out of the total treatment amount were indicated in the submitted insurance claim statement. They are expressed as Korean Won (1,000 KRW).

**Table 8:** Comparison of subgroup *t* related to type of medicine.

Type	Hospital type	Doctors' fees	Admission costs	Medication costs	Procedural costs	Anesthesia costs	Psychotherapy costs	Psychiatric costs	Injection costs/procedural costs	Laboratory costs	Radiotherapy costs	Total
TM	Tertiary Hospital	(11.96)	(2.36)	(6.43)	(7.60)	(2.34)	(3.56)	(.01)	(15.63)	(30.35)	(19.76)	(100)
	General Hospital	(17.14)	(2.52)	(9.43)	(6.95)	(1.87)	(8.68)	(.01)	(15.35)	(21.44)	(16.61)	(100)
	Hospital	(17.52)	(2.44)	(5.93)	(6.24)	(2.56)	(14.34)	(.00)	(17.23)	(19.82)	(13.94)	(100)
	Long-term care hospital	(23.67)	(3.96)	(3.39)	(.76)	(.87)	(17.46)	(.00)	(44.73)	(1.85)	(3.31)	(100)
	Western Clinic	(31.99)	(.18)	(1.29)	(2.58)	(3.54)	(35.12)	(.00)	(16.82)	(1.98)	(6.50)	(100)
	Dental Hospital	(17.57)	(.00)	(.42)	(.00)	(.00)	(.00)	(.00)	(82.01)	(.00)	(.00)	(100)
	Public Health Center	(.00)	(.00)	(.08)	(1.48)	(.00)	(96.83)	(.00)	(.35)	(1.21)	(.03)	(100)
	Local Public Health Clinic	(.00)	(.00)	(49.13)	(.34)	(.00)	(47.29)	(.00)	(2.68)	(.55)	(.00)	(100)
	Public Health Hospital	(35.19)	(.20)	(4.47)	(2.06)	(.16)	(18.89)	(.00)	(29.61)	(2.62)	(6.79)	(100)

WM	Traditional Hospital	(17.99)	(3.57)	(2.61)	(.08)	(.46)	(1.44)	(.00)	(68.04)	(4.17)	(1.64)	(100)
	Traditional Clinic	(21.61)	(.01)	(6.44)	(.00)	(.09)	(.00)	(.00)	(71.86)	(.00)	(.00)	(100)

	Total	(25.94)	(.59)	(3.89)	(2.52)	(2.25)	(20.08)	(.00)	(33.67)	(4.95)	(6.10)	(100)

WM, Western medicine; TM, Korean traditional medicine.

**Table 9:** Comparison of lesion frequency by type of medical practice in 2011–2014.

Class	Year	Type	Knee lesions [M17]			Shoulder lesions [M75]			Wrist and hand level [S63]			Ankle and foot level [S93]		
			Growth^†^	*N* ^*∗*^	%	Growth^†^	*N* ^*∗*^	%	Growth^†^	*N* ^*∗*^	%	Growth^†^	*N* ^*∗*^	%
H	2011	WM		5,024	97.97		2,399	98.36		216	97.74		860	94.82
		TM		104	2.03		40	1.64		5	2.26		47	5.18
		Total		5,128	100.00		2,439	100.00		221	100.00		907	100.00
	2012	WM	25.72	6,316	96.74	27.97	3,070	97.90	−5.56	204	97.61	12.79	970	96.33
		TM	104.81	213	3.26	65.00	66	2.10	0.00	5	2.39	−21.28	37	3.67
		Total	27.32	6,529	100.00	28.58	3,136	100.00	−5.43	209	100.00	11.03	1,007	100.00
	2013	WM	41.04	7,086	96.45	53.61	3,685	98.03	21.76	263	98.50	30.00	1,118	94.59
		TM	150.96	261	3.55	85.00	74	1.97	−20.00	4	1.50	36.17	64	5.41
		Total	43.27	7,347	100.00	54.12	3,759	100.00	20.81	267	100.00	30.32	1,182	100.00
	2014	WM	58.04	7,940	95.62	77.99	4,270	98.12	17.13	253	98.44	50.70	1,296	94.81
		TM	250.00	364	4.38	105.00	82	1.88	−20.00	4	1.56	51.06	71	5.19
		Total	61.93	8,304	100.00	78.43	4,352	100.00	16.29	257	100.00	50.72	1,367	100.00

O	2011	WM		508,830	79.13		258,936	58.24		83,624	61.16		123,147	49.65
		TM		134,181	20.87		185,699	41.76		53,106	38.84		124,891	50.35
		Total		643,011	100.00		444,635	100.00		136,730	100.00		248,038	100.00
	2012	WM	5.41	536,381	79.33	14.91	297,536	63.60	5.76	88,439	61.49	7.63	132,544	50.85
		TM	4.18	139,790	20.67	−8.31	170,259	36.40	4.31	55,394	38.51	2.56	128,092	49.15
		Total	5.16	676,171	100.00	5.21	467,795	100.00	5.19	143,833	100.00	5.08	260,636	100.00
	2013	WM	7.14	545,161	78.94	17.50	304,245	64.47	7.08	89,546	59.56	11.24	136,983	50.96
		TM	8.37	145,411	21.06	−9.73	167,639	35.53	14.49	60,801	40.44	5.53	131,801	49.04
		Total	7.40	690,572	100.00	6.13	471,884	100.00	9.96	150,347	100.00	8.36	268,784	100.00
	2014	WM	9.18	555,535	79.63	17.99	305,510	65.84	9.83	91,843	60.39	13.77	140,107	51.52
		TM	5.90	142,092	20.37	−16.45	155,153	0.72	13.46	60,252	39.61	5.55	131,824	48.48
		Total	8.49	697,627	100.00	4.35	463,993	100.00	11.24	152,095	100.00	9.63	271,931	100.00

^*∗*^Patients with overlapping records were tallied as one patient (overlap was not allowed). ^†^Growth rate compared to 2011. H, hospitalization; O, outpatient; WM, Western medicine; TM, traditional Korean medicine.

**Table 10:** Numbers of hospitalizations for gonarthrosis [M17] patients by hospital type.

Year	Type of medicine	Hospital type	Frequency		Hospitalization costs			LOS
			*N* ^*∗*^	%	Cost^†^	%	Per diem^‡^	Days per episode^§^
2011	Western	Tertiary Hospital	204	3.98	1,225	9.50	460,420	14.20
		General Hospital	589	11.49	3,215	24.93	254,225	22.53
		Hospital	1,689	32.94	7,044	54.62	225,222	17.68
		Long-term care hospital	1,857	36.21	553	4.29	94,819	4.42
		Western Clinic	683	13.32	794	6.16	81,749	13.64
		Public Health Hospital	2	0.04	1	0.01	39,420	11.00
		Total	5,024	97.97	12,832	99.50	170,393	12.66
	Traditional	Traditional Hospital	99	1.93	62	0.48	57,349	13.10
		Traditional Clinic	5	0.10	2	0.02	33,235	13.80
		Total	104	2.03	64	0.50	56,190	13.13
	Total		5,128	100.00	12,896	100.00	168,077	12.67

2012	Western	Tertiary Hospital	187	2.86	1,190	8.81	490,276	14.06
		General Hospital	615	9.42	3,188	23.60	236,169	22.07
		Hospital	2,011	30.80	7,435	55.05	212,520	15.80
		Long-term care hospital	2,792	42.76	700	5.18	99,643	3.50
		Western Clinic	710	10.87	868	6.42	78,343	13.69
		Public Health Hospital	1	0.02	1	0.01	42,477	19.00
		Total	6,316	96.74	13,383	99.08	158,039	10.69
	Traditional	Traditional Hospital	190	2.91	114	0.84	62,827	12.07
		Traditional Clinic	23	0.35	11	0.08	33,646	15.57
		Total	213	3.26	125	0.92	59,676	12.45
	Total		6,529	100.00	13,507	100.00	154,830	10.74

2013	Western	Tertiary Hospital	195	2.65	1,287	8.79	454,263	16.49
		General Hospital	725	9.87	3,681	25.16	234,228	21.27
		Hospital	1,997	27.18	7,678	52.49	215,432	16.46
		Long-term care hospital	3,393	46.18	888	6.07	98,884	3.62
		Western Clinic	774	10.53	939	6.42	84,784	12.97
		Public Health Hospital	2	0.03	1	0.01	44,311	16.00
		Total	7,086	96.45	14,474	98.95	153,802	10.42
	Traditional	Traditional Hospital	234	3.18	140	0.96	64,218	11.45
		Traditional Clinic	27	0.37	14	0.10	42,487	13.56
		Total	261	3.55	154	1.05	61,970	11.67
	Total		7,347	100.00	14,628	100.00	150,539	10.47

2014	Western	Tertiary Hospital	272	3.28	1,641	11.46	459,732	14.81
		General Hospital	727	8.75	3,723	26.01	233,047	22.00
		Hospital	2,152	25.92	6,911	48.29	205,374	14.52
		Long-term care hospital	4,022	48.43	1,045	7.30	102,035	3.44
		Western Clinic	766	9.22	783	5.47	75,945	13.00
		Public Health Hospital	1	0.01	0	0.00	63,640	4.00
		Total	7,940	95.62	14,103	98.54	151,771	9.46
	Traditional	Traditional Hospital	329	3.96	193	1.35	64,804	11.00
		Traditional Clinic	35	0.42	16	0.11	38,910	13.77
		Total	364	4.38	209	1.46	62,314	11.26
	Total		8,304	100.00	14,312	100.00	147,849	9.54

^*∗*^Patients with overlapping records were tallied as one patient (overlap was not allowed). ^†^Costs determined to be eligible for reimbursement by the HIRA (Health Insurance Review and Assessment Service) out of the total treatment amount were indicated in the submitted insurance claim statement. They are expressed as means and are in Korean Won (1,000,000 KRW). ^‡^Per diem is the average daily cost of services covered by National Health Insurance. ^§^Days per episode are the total number of hospitalized days divided by the total number of hospitalizations.

**Table 11:** Number of outpatients with gonarthrosis [M17] by hospital type.

Year	Type of medicine	Hospital type	Frequency		Outpatient costs			LOS
			*N* ^*∗*^	%	Cost^†^	%	Per diem^‡^	Days per episode^§^
2011	Western	Tertiary Hospital	3,771	0.59	199	1.35	39,490	1.76
		General Hospital	13,999	2.18	689	4.69	32,279	6.26
		Hospital	28,259	4.39	1,667	11.33	28,169	2.04
		Long-term care hospital	1,872	0.29	95	0.65	15,504	3.51
		Clinic	458,027	71.23	9,641	65.55	21,049	1.02
		Public Health Center	1,495	0.23	5	0.04	3,474	1.00
		Local Public Health Clinic	389	0.06	5	0.03	12,991	7.70
		Public Health Hospital	1,018	0.16	27	0.18	26,723	1.67
		Total	508,830	79.13	12,329	83.83	21,823	1.24
	Traditional	Traditional Hospital	1,022	0.16	49	0.33	16,099	2.81
		Traditional Clinic	133,159	20.71	2,330	15.84	17,497	1.04
		Total	134,181	20.87	2,379	16.17	17,486	1.05
	Total		643,011	100.00	14,707	100.00	20,918	1.20

2012	Western	Tertiary Hospital	4,817	0.71	215	1.42	44,547	1.55
		General Hospital	20,949	3.10	720	4.76	34,367	4.58
		Hospital	54,982	8.13	1,858	12.27	33,789	1.12
		Long-term care hospital	4,519	0.67	99	0.66	22,003	1.42
		Clinic	448,226	66.29	9,691	64.01	21,621	1.02
		Public Health Center	1,592	0.00	6	0.00	3,811	1.01
		Local Public Health Clinic	439	0.24	3	0.04	7,361	4.12
		Public Health Hospital	855	0.06	26	0.02	30,431	1.46
		Dental Hospital	2	0.13	0	0.17	24,970	1.00
		Total	536,381	79.33	12,618	83.34	23,525	1.18
	Traditional	Traditional Hospital	2,332	0.34	50	0.33	21,369	1.16
		Traditional Clinic	137,458	20.33	2,472	16.33	17,986	1.03
		Total	139,790	20.67	2,522	16.66	18,042	1.03
	Total		676,171	100.00	15,140	100.00	22,391	1.15

2013	Western	Tertiary Hospital	4,949	0.72	218	1.37	44,048	1.57
		General Hospital	21,512	3.12	783	4.92	36,416	4.20
		Hospital	55,199	7.99	1,879	11.81	34,034	1.12
		Long-term care hospital	4,780	0.69	104	0.66	21,854	1.49
		Clinic	456,160	66.06	10,203	64.13	22,367	1.01
		Public Health Center	1,678	0.24	6	0.04	3,442	1.05
		Local Public Health Clinic	191	0.03	2	0.02	12,633	6.90
		Public Health Hospital	692	0.10	23	0.15	33,579	1.97
		Total	545,161	78.94	13,219	83.09	24,248	1.16
	Traditional	Traditional Hospital	2,306	0.33	52	0.32	22,375	1.13
		Traditional Clinic	143,105	20.72	2,639	16.59	18,443	1.03
		Total	145,411	21.06	2,691	16.91	18,505	1.03
	Total		690,572	100.00	15,910	100.00	23,039	1.14

2014	Western	Tertiary Hospital	5,377	0.77	243	1.43	45,133	1.24
		General Hospital	22,999	3.30	856	5.06	37,234	3.85
		Hospital	57,441	8.23	1,986	11.74	34,571	1.11
		Long-term care hospital	4,748	0.68	106	0.63	22,320	1.48
		Clinic	461,684	66.18	10,962	64.81	23,742	1.01
		Public Health Center	2,372	0.00	8	0.00	3,298	1.00
		Local Public Health Clinic	171	0.34	2	0.05	12,230	6.67
		Public Health Hospital	732	0.02	21	0.01	28,470	1.31
		Dental Hospital	11	0.10	0	0.12	18,469	1.00
		Total	555,535	79.63	14,183	83.86	25,531	1.15
	Traditional	Traditional Hospital	2,516	0.36	59	0.35	23,541	1.13
		Traditional Clinic	139,576	20.01	2,670	15.79	19,127	1.03
		Total	142,092	20.37	2,729	16.14	19,205	1.03
	Total		697,627	100.00	16,912	100.00	24,242	1.12

^*∗*^Patients with overlapping records were tallied as one patient (overlap was not allowed). ^†^Costs determined to be eligible for reimbursement by the HIRA (Health Insurance Review and Assessment Service) out of the total treatment amount were indicated in the submitted insurance claim statement. They is expressed as a mean and are in Korean Won (1,000,000 KRW). ^‡^Per diem is the average daily cost of services covered by National Health Insurance. ^§^Days per episode are the total number of outpatient visit days including drug prescription days divided by the total number of outpatient visits.

**Table 12:** Number of hospitalizations for shoulder lesion [M75] patients by hospital type.

Year	Type of medicine	Hospital type	Frequency		Hospitalization costs			LOS
			*N* ^*∗*^	%	Cost^†^	%	Per diem^‡^	Days per episode^§^
2011	Western	Tertiary Hospital	135	5.54	278	9.55	377,378	6.08
		General Hospital	381	15.62	765	26.31	210,523	12.83
		Hospital	1,101	45.14	1,608	55.27	203,071	8.86
		Long-term care hospital	589	24.15	69	2.37	94,749	1.45
		Western Clinic	193	7.91	167	5.74	99,580	11.10
		Total	2,399	98.36	2,886	99.24	179,142	7.69
	Traditional	Traditional Hospital	39	1.6	22	0.75	53203	14.26
		Traditional Clinic	1	0.04	0	0.01	21440	14
		Total	40	1.64	22	0.76	52409	14.25
	Total		2,439	100.00	2,908	100.00	177,064	7.80

2012	Western	Tertiary Hospital	142	4.53	318	8.32	374,833	6.37
		General Hospital	468	14.92	989	25.85	208,846	12.43
		Hospital	1,547	49.33	2,168	56.66	200,108	8.36
		Long-term care hospital	661	21.08	75	1.96	98,519	1.25
		Western Clinic	252	8.04	242	6.33	138,525	9.81
		Total	3,070	97.90	3,792	99.13	182,594	7.48
	Traditional	Traditional Hospital	62	1.98	32	0.84	58,945	11.53
		Traditional Clinic	4	0.13	1	0.03	31,056	14.25
		Total	66	2.10	33	0.87	57,255	11.70
	Total		3,136	100.00	3,826	100.00	179,956	7.57

2013	Western	Tertiary Hospital	169	4.50	388	8.65	379,396	6.41
		General Hospital	582	15.48	1,212	27.01	188,152	13.07
		Hospital	1,920	51.08	2,524	56.27	193,851	7.58
		Long-term care hospital	739	19.66	100	2.22	95,331	1.68
		Western Clinic	274	7.29	223	4.97	156,193	8.29
		Public Health Hospital	1	0.03	0	0.00	89,370	1.00
		Total	3,685	98.03	4,446	99.12	178,875	7.26
	Traditional	Traditional Hospital	68	1.81	37	0.82	64,497	10.57
		Traditional Clinic	6	0.16	3	0.06	40,844	14.17
		Total	74	1.97	40	0.88	62,579	10.86
	Total		3,759	100.00	4,485	100.00	176,585	7.33

2014	Western	Tertiary Hospital	161	3.70	333	6.97	377,875	5.68
		General Hospital	671	15.42	1,214	25.45	202,746	10.41
		Hospital	2,298	52.80	2,773	58.12	188,633	6.86
		Long-term care hospital	817	18.77	134	2.80	99,291	2.07
		Western Clinic	323	7.42	276	5.79	164,902	8.50
		Total	4,270	98.12	4,730	99.12	179,097	6.58
	Traditional	Traditional Hospital	76	1.75	39	0.83	60,405	10.09
		Traditional Clinic	6	0.14	2	0.05	23,633	17.17
		Total	82	1.88	42	0.88	57,714	10.61
	Total		4,352	100.00	4,772	100.00	176,810	6.66

^*∗*^Patients with overlapping records were tallied as one patient (overlap not allowed). ^†^Costs determined to be eligible for reimbursement by the HIRA (Health Insurance Review and Assessment Service) out of the total treatment amount were indicated in the submitted insurance claim statement. They are expressed as means and are in Korean Won (1,000,000 KRW). ^‡^Per diem is the average daily cost of services covered by National Health Insurance. ^§^Days per episode are the total number of hospitalized days divided by the total number of hospitalizations.

**Table 13:** Number of outpatients with shoulder lesions [M75] by hospital type.

Year	Type of medicine	Hospital type	Frequency		Outpatient costs			LOS
			*N* ^*∗*^	%	Cost^†^	%	Per diem^‡^	Days per episode^§^
2011	Western	Tertiary Hospital	3,279	0.74	147	1.55	29,079	2.14
		General Hospital	8,754	1.97	420	4.42	26,730	4.09
		Hospital	16,434	3.70	803	8.45	21,712	2.21
		Long-term care hospital	1,120	0.25	47	0.50	16,906	2.84
		Clinic	228,892	51.48	4,559	47.99	19,916	1.01
		Dental Hospital	8	0.00	0	0.00	18,765	1.63
		Public Health Center	301	0.07	1	0.01	3,204	1.00
		Local Public Health Clinic	22	0.00	0	0.00	5,281	1.91
		Public Health Hospital	126	0.03	3	0.03	23,603	1.25
		Total	258,936	58.24	5,980	62.96	20,345	1.21
	Traditional	Traditional Hospital	1,327	0.30	62	0.66	17,637	2.58
		Traditional Clinic	184,372	41.47	3,456	36.39	18,746	1.04
		Total	185,699	41.76	3,519	37.04	18,738	1.05
	Total		444,635	100.00	9,499	100.00	19,674	1.14

2012	Western	Tertiary Hospital	4,805	1.03	150	1.54	31,203	1.33
		General Hospital	16,148	3.45	418	4.29	25,911	2.07
		Hospital	42,956	9.18	1,004	10.29	23,362	1.05
		Long-term care hospital	2,372	0.51	52	0.53	21,931	1.40
		Clinic	230,518	49.28	4,790	49.14	20,780	1.01
		Dental Hospital	5	0.00	0	0.00	26,800	1.00
		Public Health Center	552	0.12	2	0.02	3,290	1.00
		Local Public Health Clinic	12	0.00	0	0.00	9,058	3.92
		Public Health Hospital	168	0.04	4	0.04	22,856	3.33
		Total	297,536	63.60	6,420	65.85	21,577	1.08
	Traditional	Traditional Hospital	2,348	0.50	52	0.53	22,053	1.31
		Traditional Clinic	167,911	35.89	3,277	33.62	19,517	1.03
		Total	170,259	36.40	3,329	34.15	19,552	1.04
	Total		467,795	100.00	9,749	100.00	20,840	1.07

2013	Western	Tertiary Hospital	5,053	1.07	167	1.63	33,032	1.29
		General Hospital	15,611	3.31	434	4.26	27,831	2.06
		Hospital	46,514	9.86	1,108	10.85	23,823	1.05
		Long-term care hospital	2,105	0.45	49	0.48	23,288	1.45
		Clinic	234,099	49.61	5,077	49.73	21,688	1.01
		Dental Hospital	6	0.00	0	0.00	17,590	1.00
		Public Health Center	701	0.15	2	0.02	2,487	1.00
		Local Public Health Clinic	24	0.01	0	0.00	6,914	2.96
		Public Health Hospital	132	0.03	3	0.03	25,864	1.74
		Total	304,245	64.47	6,841	67.01	22,486	1.08
	Traditional	Traditional Hospital	2,163	0.46	51	0.50	23,727	1.09
		Traditional Clinic	165,476	35.07	3,317	32.49	20,047	1.03
		Total	167,639	35.53	3,369	32.99	20,095	1.03
	Total		471,884	100.00	10,210	100.00	21,636	1.06

2014	Western	Tertiary Hospital	4,474	0.96	149	1.42	33,281	1.34
		General Hospital	17,733	3.82	502	4.79	28,320	2.11
		Hospital	48,171	10.38	1,136	10.85	23,590	1.04
		Long-term care hospital	2,106	0.45	50	0.47	23,507	1.36
		Clinic	232,309	50.07	5,315	50.74	22,881	1.01
		Public Health Center	471	0.10	2	0.02	3,848	1.37
		Local Public Health Clinic	35	0.01	0	0.00	7,430	3.80
		Public Health Hospital	211	0.05	6	0.06	28,187	1.34
		Total	305,510	65.84	7,160	68.35	23,437	1.09
	Traditional	Traditional Hospital	3,330	33.44	73	30.96	21,781	1.03
		Traditional Clinic	155,153	0.72	3,243	0.69	20,903	1.03
		Total	158,483	34.16	3,316	31.65	20,921	1.03
	Total		463,993	100.00	10,476	100.00	22,578	1.07

^*∗*^Patients with overlapping records were tallied as one patient (overlap was not allowed). ^†^Costs determined to be eligible for reimbursement by the HIRA (Health Insurance Review and Assessment Service) out of the total treatment amount were indicated in the submitted insurance claim statement. They are expressed as means and are in Korean Won (1,000,000 KRW). ^‡^Per diem is the average daily cost of services covered by National Health Insurance. ^§^Days per episode are the total number of outpatient visit days including drug prescription days divided by the total number of outpatient visits.

**Table 14:** Number of hospitalizations for wrist and hand level lesions [S63] by hospital type.

Year	Type of medicine	Hospital type	Frequency		Hospitalization costs			LOS
			*N* ^*∗*^	%	Cost^†^	%	Per diem^‡^	Days per episode^§^
2011	Western	Tertiary Hospital	11	4.98	13	7.68	278,227	5.45
		General Hospital	45	20.36	43	25.65	179,511	6.20
		Hospital	97	43.89	74	44.40	139,886	6.36
		Long-term care hospital	11	4.98	1	0.84	61,642	2.18
		Western Clinic	52	23.53	34	20.56	82,895	9.83
		Total	216	97.74	165	99.13	137,482	6.90
	Traditional	Traditional Hospital	4	1.81	1	0.59	50,891	4.75
		Traditional Clinic	1	0.45	0	0.28	32,616	14.00
		Total	5	2.26	1	0.87	47,236	6.60
	Total		221	100.00	166	100.00	135,440	6.90

2012	Western	Tertiary Hospital	9	4.31	10	6.32	248,187	4.89
		General Hospital	32	15.31	31	20.03	181,560	8.19
		Hospital	112	53.59	87	55.96	127,213	7.51
		Long-term care hospital	13	6.22	1	0.83	99,160	1.00
		Western Clinic	38	18.18	25	15.80	91,169	9.32
		Total	204	97.61	154	98.93	132,573	7.42
	Traditional	Traditional Hospital	2	0.96	1	0.61	53,661	8.00
		Traditional Clinic	3	1.44	1	0.46	41,900	7.33
		Total	5	2.39	2	1.07	46,604	7.60
	Total		209	100.00	156	100.00	130,517	7.43

2013	Western	Tertiary Hospital	11	4.12	13	7.25	342,630	3.73
		General Hospital	50	18.73	47	26.27	156,802	6.76
		Hospital	111	41.57	85	46.85	130,977	6.95
		Long-term care hospital	44	16.48	4	1.98	80,871	1.09
		Western Clinic	47	17.60	30	16.67	88,549	10.30
		Total	263	98.50	179	99.03	128,774	6.40
	Traditional	Traditional Hospital	4	1.50	2	0.97	53,481	8.00
		Total	4	1.50	2	0.97	53,481	8.00
	Total		267	100.00	180	100.00	127,646	6.42

2014	Western	Tertiary Hospital	13	5.06	12	5.82	210,379	4.00
		General Hospital	65	25.29	71	33.81	167,548	8.48
		Hospital	118	45.91	97	46.30	137,949	6.69
		Long-term care hospital	14	5.45	1	0.54	81,609	1.00
		Western Clinic	43	16.73	27	12.62	88,134	8.91
		Total	253	98.44	208	99.08	137,691	7.08
	Traditional	Traditional Hospital	3	1.17	1	0.59	64,672	8.00
		Traditional Clinic	1	0.39	1	0.33	36,168	19.00
		Total	4	1.56	2	0.92	57,546	10.75
	Total		257	100.00	210	100.00	136,444	7.13

^*∗*^Patients with overlapping records were tallied as one patient (overlap was not allowed). ^†^Costs determined to be eligible for reimbursement by the HIRA (Health Insurance Review and Assessment Service) out of the total treatment amount were indicated in the submitted insurance claim statement. They are expressed as means and are in Korean Won (1,000,000 KRW). ^‡^Per diem is the average daily cost of services covered by National Health Insurance. ^§^Days per episode are the total number of hospitalized days divided by the total number of hospitalizations.

**Table 15:** Number of outpatients for wrist and hand level lesions [S63] by hospital type.

Year	Type of medicine	Hospital type	Frequency		Outpatient costs			LOS
			*N* ^*∗*^	%	Cost^†^	%	Per diem^‡^	Days per episode^§^
2011	Western	Tertiary Hospital	251	0.18	19	0.60	58,745	1.58
		General Hospital	2,612	1.91	155	4.95	44,742	1.62
		Hospital	7,082	5.18	319	10.18	29,974	1.58
		Long-term care hospital	321	0.23	12	0.37	18,678	1.99
		Clinic	73,276	53.59	1,618	51.67	22,082	1.00
		Public Health Center	39	0.03	0	0.01	5,699	1.00
		Local Public Health Clinic	13	0.01	0	0.01	18,520	11.46
		Public Health Hospital	30	0.02	1	0.02	18,767	1.23
		Total	83,624	61.16	2,124	67.81	23,546	1.08
	Traditional	Traditional Hospital	545	0.40	21	0.68	18,048	2.18
		Traditional Clinic	52,561	38.44	987	31.51	18,777	1.03
		Total	53,106	38.84	1,008	32.19	18,770	1.04
	Total		136,730	100.00	3,132	100.00	21,691	1.06

2012	Western	Tertiary Hospital	306	0.21	16	0.48	51,987	1.36
		General Hospital	3,404	2.37	146	4.41	42,810	1.19
		Hospital	11,477	7.98	357	10.80	31,071	1.04
		Long-term care hospital	667	0.46	13	0.40	19,954	1.10
		Clinic	72,525	50.42	1,680	50.86	23,163	1.01
		Public Health Center	1	0.00	0	0.00	5,790	1.00
		Local Public Health Clinic	13	0.01	0	0.00	8,726	4.00
		Public Health Hospital	46	0.03	1	0.04	29,252	1.43
		Total	88,439	61.49	2,213	66.99	25,022	1.02
	Traditional	Traditional Hospital	838	0.58	18	0.53	20,947	1.05
		Traditional Clinic	54,556	37.93	1,073	32.48	19,664	1.02
		Total	55,394	38.51	1,090	33.01	19,683	1.02
	Total		143,833	100.00	3,303	100.00	22,966	1.02

2013	Western	Tertiary Hospital	297	0.20	19	0.54	65,123	1.40
		General Hospital	3,512	2.34	163	4.58	46,354	1.19
		Hospital	10,971	7.30	360	10.13	32,801	1.07
		Long-term care hospital	484	0.32	10	0.28	20,811	1.07
		Clinic	74,188	49.34	1,758	49.50	23,690	1.00
		Public Health Center	34	0.00	0	0.00	3,086	1.00
		Local Public Health Clinic	7	0.02	0	0.00	7,674	3.00
		Public Health Hospital	52	0.00	1	0.00	24,083	1.23
		Dental Hospital	1	0.03	0	0.04	24,540	1.00
		Total	89,546	59.56	2,311	65.09	25,808	1.02
	Traditional	Traditional Hospital	956	0.64	22	0.62	22,855	1.05
		Traditional Clinic	59,845	39.80	1,218	34.30	20,350	1.02
		Total	60,801	40.44	1,240	34.91	20,390	1.02
	Total		150,347	100.00	3,551	100.00	23,617	1.02

2014	Western	Tertiary Hospital	332	0.22	21	0.57	63,661	1.34
		General Hospital	3,886	2.55	180	4.80	46,221	1.44
		Hospital	11,366	7.47	372	9.96	32,755	1.05
		Long-term care hospital	646	0.42	14	0.38	21,995	1.20
		Clinic	75,535	49.66	1,868	49.94	24,725	1.00
		Public Health Center	10	0.00	0	0.00	4,050	1.00
		Local Public Health Clinic	14	0.01	0	0.00	8,487	3.43
		Public Health Hospital	50	0.01	1	0.00	24,903	1.18
		Dental Hospital	4	0.03	0	0.03	19,768	1.00
		Total	91,843	60.39	2,456	65.69	26,745	1.03
	Traditional	Traditional Hospital	892	0.59	20	0.55	22,893	1.08
		Traditional Clinic	59,360	39.03	1,263	33.77	21,273	1.02
		Total	60,252	39.61	1,283	34.31	21,297	1.02
	Total		152,095	100.00	3,740	100.00	24,587	1.03

^*∗*^Patients with overlapping records were tallied as one patient (overlap was not allowed). ^†^Costs determined to be eligible for reimbursement by the HIRA (Health Insurance Review and Assessment Service) out of the total treatment amount were indicated in the submitted insurance claim statement. They are expressed as means and are in Korean Won (1,000,000 KRW). ^‡^Per diem is the average daily cost of services covered by National Health Insurance. ^§^Days per episode are the total number of outpatient visit days including drug prescription days divided by the total number of outpatient visits.

**Table 16:** Number of hospitalizations for ankle and foot level lesions [S93] by hospital type.

Year	Type of medicine	Hospital type	Frequency		Hospitalization costs			LOS
			*N* ^*∗*^	%	Cost^†^	%	Per diem^‡^	Days per episode^§^
2011	Western	Tertiary Hospital	5	0.55	8	1.24	173,270	10.40
		General Hospital	108	11.91	123	18.23	98,380	12.77
		Hospital	345	38.04	280	41.41	84,468	10.59
		Long-term care hospital	34	3.75	6	0.96	65,373	3.26
		Western Clinic	368	40.57	241	35.54	56,559	12.45
		Total	860	94.82	659	97.38	74,034	11.37
	Traditional	Traditional Hospital	42	4.63	17	2.47	49,012	9.38
		Traditional Clinic	5	0.55	1	0.15	34,441	6.80
		Total	47	5.18	18	2.62	47,462	9.11
	Total		907	100.00	677	100.00	72,657	11.25

2012	Western	Tertiary Hospital	10	0.99	12	1.61	193,439	6.00
		General Hospital	148	14.70	156	20.70	108,791	11.49
		Hospital	391	38.83	305	40.51	88,346	9.74
		Long-term care hospital	38	3.77	12	1.61	82,398	5.34
		Western Clinic	383	38.03	250	33.20	59,570	11.50
		Total	970	96.33	734	97.63	80,954	10.49
	Traditional	Traditional Hospital	32	3.18	16	2.17	46,740	12.50
		Traditional Clinic	5	0.50	2	0.20	32,100	9.80
		Total	37	3.67	18	2.37	44,761	12.14
	Total		1,007	100.00	752	100.00	79,624	10.55

2013	Western	Tertiary Hospital	8	0.68	13	1.41	206,721	7.75
		General Hospital	186	15.74	192	21.49	117,806	9.83
		Hospital	465	39.34	390	43.78	104,115	8.94
		Long-term care hospital	61	5.16	14	1.57	93,890	3.52
		Western Clinic	398	33.67	262	29.42	64,569	11.39
		Total	1,118	94.59	871	97.67	92,491	9.65
	Traditional	Traditional Hospital	60	5.08	20	2.23	66,044	6.93
		Traditional Clinic	4	0.34	1	0.11	22,968	11.50
		Total	64	5.41	21	2.33	63,352	7.22
	Total		1,182	100.00	891	100.00	90,913	9.52

2014	Western	Tertiary Hospital	9	0.66	10	0.92	200,250	6.00
		General Hospital	214	15.65	234	22.08	127,396	10.31
		Hospital	625	45.72	533	50.31	105,591	8.72
		Long-term care hospital	72	5.27	24	2.27	92,879	5.67
		Western Clinic	376	27.51	235	22.22	63,569	10.69
		Total	1,296	94.81	1,036	97.81	96,951	9.36
	Traditional	Traditional Hospital	61	4.46	20	1.90	58,725	7.28
		Traditional Clinic	10	0.73	3	0.29	25,945	12.80
		Total	71	5.19	23	2.19	54,108	8.06
	Total		1,367	100.00	1,059	100.00	94,726	9.30

^*∗*^Patients with overlapping records were tallied as one patient (overlap not allowed). ^†^Costs determined to be eligible for reimbursement by the HIRA (Health Insurance Review and Assessment Service) out of the total treatment amount were indicated in the submitted insurance claim statement. They are expressed as means and are in Korean Won (1,000,000 KRW). ^‡^Per diem is the average daily cost of services covered by National Health Insurance. ^§^Days per episode are the total number of hospitalized days divided by the total number of hospitalizations.

**Table 17:** Number of outpatient visits for ankle and foot level lesions [S93] by hospital type.

Year	Type of medicine	Hospital type	Frequency		Outpatient costs			LOS
			*N* ^*∗*^	%	Cost^†^	%	Per diem^‡^	Days per episode^§^
2011	Western	Tertiary Hospital	559	0.23	46	0.79	71,456	1.84
		General Hospital	4,204	1.69	285	4.88	54,910	1.60
		Hospital	10,466	4.22	571	9.77	35,586	1.69
		Long-term care hospital	465	0.19	18	0.31	23,088	1.90
		Clinic	107,340	43.28	2,471	42.30	23,025	1.00
		Dental Hospital	6	0.00	0	0.01	16,350	2.67
		Public Health Center	10	0.00	0	0.00	3,012	1.00
		Local Public Health Clinic	18	0.01	0	0.00	7,036	2.56
		Public Health Hospital	79	0.03	2	0.04	31,605	1.52
		Total	123,147	49.65	3,395	58.10	25,402	1.09
	Traditional	Traditional Hospital	1,141	0.46	45	0.78	18,915	2.03
		Traditional Clinic	123,750	49.89	2,403	41.12	19,416	1.03
		Total	124,891	50.35	2,448	41.90	19,412	1.04
	Total		248,038	100.00	5,843	100.00	22,386	1.06

2012	Western	Tertiary Hospital	769	0.30	50	0.80	64,899	1.51
		General Hospital	5,806	2.23	310	5.00	53,426	1.27
		Hospital	17,632	6.76	625	10.07	35,457	1.06
		Long-term care hospital	903	0.35	19	0.31	21,587	1.12
		Clinic	107,284	41.16	2,589	41.71	24,132	1.00
		Dental Hospital	31	0.01	1	0.01	26,883	1.00
		Public Health Center	14	0.01	0	0.00	4,871	1.00
		Local Public Health Clinic	23	0.01	0	0.00	9,318	4.30
		Public Health Hospital	82	0.03	3	0.04	31,415	1.39
		Total	132,544	50.85	3,597	57.96	27,142	1.03
	Traditional	Traditional Hospital	2,414	0.93	54	0.86	22,190	1.06
		Traditional Clinic	125,678	48.22	2,556	41.18	20,336	1.03
		Total	128,092	49.15	2,609	42.04	20,371	1.03
	Total		260,636	100.00	6,207	100.00	23,814	1.03

2013	Western	Tertiary Hospital	752	0.28	50	0.76	66,703	1.68
		General Hospital	6,417	2.39	357	5.40	55,586	1.22
		Hospital	18,960	7.05	675	10.22	35,603	1.05
		Long-term care hospital	773	0.29	17	0.25	21,454	1.10
		Clinic	109,956	40.91	2,731	41.35	24,835	1.00
		Dental Hospital	4	0.00	0	0.00	25,545	1.00
		Public Health Center	28	0.01	0	0.00	2,908	1.00
		Local Public Health Clinic	23	0.01	0	0.00	6,697	2.83
		Public Health Hospital	70	0.03	2	0.03	30,855	1.23
		Total	136,983	50.96	3,832	58.02	27,972	1.02
	Traditional	Traditional Hospital	2,255	0.84	52	0.79	23,228	1.05
		Traditional Clinic	129,546	48.20	2,720	41.18	20,994	1.02
		Total	131,801	49.04	2,772	41.98	21,032	1.02
	Total		268,784	100.00	6,604	100.00	24,569	1.02

2014	Western	Tertiary Hospital	634	0.23	53	0.76	83,468	1.66
		General Hospital	7,410	2.72	415	5.95	56,052	1.27
		Hospital	19,690	7.24	715	10.25	36,321	1.05
		Long-term care hospital	747	0.27	17	0.25	23,249	1.08
		Clinic	111,528	41.01	2,884	41.34	25,857	1.00
		Dental Hospital	3	0.00	0	0.00	19,330	1.00
		Public Health Center	12	0.00	0	0.00	4,125	1.00
		Local Public Health Clinic	16	0.01	0	0.00	10,259	4.63
		Public Health Hospital	67	0.02	2	0.03	28,492	1.03
		Total	140,107	51.52	4,087	58.58	29,169	1.03
	Traditional	Traditional Hospital	2,080	0.76	51	0.73	24,517	1.04
		Traditional Clinic	129,744	47.71	2,839	40.69	21,880	1.02
		Total	131,824	48.48	2,890	41.42	21,922	1.02
	Total		271,931	100.00	6,977	100.00	25,656	1.03

^*∗*^Patients with overlapping records were tallied as one patient (overlap not allowed). ^†^Costs determined to be eligible for reimbursement by the HIRA (Health Insurance Review and Assessment Service) out of the total treatment amount were indicated in the submitted insurance claim statement. They are expressed as means and are in Korean Won (1,000,000 KRW). ^‡^Per diem is the average daily cost of services covered by National Health Insurance. ^§^Days per episode are the total number of outpatient visit days including drug prescription days divided by the total number of outpatient visits.

**Table 18:** Distribution of nonsurgical interventions in Western medicine and traditional Korean medicine.

Nonsurgical intervention		Total	*Year*	*2011*	*2012*	*2013*	*2014*
			*N* ^*∗*^	1,481,969	1,560,032	1,594,949	1,600,774
WM	Basic physical therapy^†^	0	*N*	947,982	1,000,863	1,034,024	1,033,566
			%	(63.97)	(64.16)	(64.83)	(64.57)
		1	*N*	32,582	38,311	46,856	45,063
			%	(2.20)	(2.46)	(2.94)	(2.82)
		2	*N*	136,773	137,871	126,126	122,500
			%	(9.23)	(8.84)	(7.91)	(7.65)
		3≦	N	364,632	382,987	387,943	399,645
			%	(24.60)	(24.55)	(24.32)	(24.97)

WM	Simple rehabilitation^‡^	0	*N*	1,475,271	1,551,724	1,585,834	1,590,708
			%	(99.55)	(99.47)	(99.43)	(99.37)
		1≦	*N*	6,698	8,308	9,115	10,066
			%	(.45)	(.53)	(.57)	(.63)

WM	Professional rehabilitation^§^	0	*N*	1,481,873	1,560,008	1,594,914	1,600,736
			%	(99.99)	(100.00)	(100.00)	(100.00)
		1≦	*N*	96	24	35	38
			%	(.01)	(.00)	(.00)	(.00)

WM	Rehabilitation of CNS	0	*N*	1,481,962	1,560,029	1,594,942	1,600,773
			%	(100.00)	(100.00)	(100.00)	(100.00)
		1≦	*N*	7	3	7	1
			%	(.00)	(.00)	(.00)	(.00)

TM	Acupuncture	0	*N*	982,471	1,062,109	1,085,224	1,102,972
			%	(66.29)	(68.08)	(68.04)	(68.90)
		1	*N*	40,678	44,984	55,124	50,447
			%	(2.74)	(2.88)	(3.46)	(3.15)
		2≦	*N*	458,820	452,939	454,601	447,355
			%	(30.96)	(29.03)	(28.50)	(27.95)

TM	Moxibustion	0	*N*	1,481,922	1,559,985	1,594,876	1,600,667
			%	(100.00)	(100.00)	(100.00)	(99.99)
		1≦	*N*	47	47	73	107
			%	(.00)	(.00)	(.00)	(.01)

TM	Cupping	0	*N*	1,481,712	1,559,844	1,594,897	1,600,518
			%	(99.98)	(99.99)	(100.00)	(99.98)
		1≦	*N*	257	188	52	256
			%	(.02)	(.01)	(.00)	(.02)

TM	Heat & cold therapy	0	*N*	1,481,856	1,559,985	1,594,897	1,600,734
			%	(99.99)	(100.00)	(100.00)	(100.00)
		1≦	*N*	113	47	52	40
			%	(.01)	(.00)	(.00)	(.00)

^*∗*^A patient could be hospitalized more than once during the study period, resulting in more than one claim per patient. Thus, the number of claims in the study was higher than the number of patients. ^†^Basic physical therapy included superficial heat therapy, cold therapy, deep heat therapy, ultraviolet irradiation, transcutaneous electrical nerve stimulation, massage therapy, and simple therapeutic exercise. ^‡^Simple rehabilitation included paraffin bath, hydrotherapy, intermittent traction therapy, electrical stimulation therapy, laser therapy, therapeutic exercise, motor point block, pneumatic compression, complex decongestive physical therapy, and iontophoresis. ^§^Professional rehabilitation included pool therapy, occupational therapy, activities of daily living training, neurogenic bladder training, functional electrical stimulation therapy, myofascial trigger point injection, rehabilitative social work, rehabilitative breathing therapy, rehabilitative functional training, and rehabilitative dysphagia therapy. WM, Western medicine; TM, traditional Korean medicine.

## Abbreviations

HIRA: Health Insurance Review & Assessment Services
NHIS: National Health Insurance Sample
WM: Western medicine
TM: Traditional Korean medicine.

## Additional Points

*Availability of Data and Material*. Datasets generated and/or analyzed in the current study are available from the HIRA-NPS repository. HIRA data are third-party data not owned by the authors. HIRA data are available upon visit or by mail upon direct, email, or fax submission of dataset request form and declaration of data use downloadable from the “HIRA” website: [http://opendata.hira.or.kr/op/opc/selectPatDataAplInfoView.do] and upon payment of the transfer of data request fee (300,000 KRW per dataset).

## References

[1] Brooks P. M. (2006). The burden of musculoskeletal disease—a global perspective. *Clinical Rheumatology*.

[2] Oh I.-H., Yoon S.-J., Seo H.-Y., Kim E.-J., Kim Y. A. (2011). The economic burden of musculoskeletal disease in Korea: A cross sectional study. *BMC Musculoskeletal Disorders*.

[3] Hur N., Choi C., Uhm W., Bae S. (2008). The Prevalence and Trend of Arthritis in Korea: Results from Korea National Health and Nutrition Examination Surveys. *The Journal of the Korean Rheumatism Association*.

[4] Choi H., Han W., Im J., Baek H. (2009). The Prevalence and Clinical Features of Musculoskeletal Diseases in Incheon: Results from Chronic Disease Management Surveys. *The Journal of the Korean Rheumatism Association*.

[5] Brage S., Nygard J. F., Tellnes G. (1997). The gender gap in musculoskeletal-related long term sickness absence in Norway. *Scandinavian Journal of Social Medicine*.

[6] Woolf A. D., Åkesson K. (2001). Understanding the burden of musculoskeletal conditions. *British Medical Journal*.

[7] Bodeker G. (2001). Lessons on integration from the developing world's experience. *British Medical Journal*.

[8] Ahn Y.-J., Shin J.-S., Lee J. (2016). Evaluation of use and cost of medical care of common lumbar disorders in Korea: Cross-sectional study of Korean Health Insurance Review and Assessment Service National Patient Sample data. *BMJ Open*.

[9] Kim J., Son M. National Health Insurance Statistical Yearbook.

[10] Badley E. M., Rasooly I., Webster G. K. (1994). Relative importance of musculoskeletal disorders as a cause of chronic health problems, disability, and health care utilization: Findings from the 1990 Ontario Health Survey. *The Journal of Rheumatology*.

[11] Coyte P. C., Asche C. V., Croxford R., Chan B. (1998). The economic cost of musculoskeletal disorders in Canada. *Arthritis & Rheumatology*.

[12] Lubeck D. P. (2003). The costs of musculoskeletal disease: Health needs assessment and health economics. *Best Practice & Research Clinical Rheumatology*.

[13] Joo H., Lee Y. J., Shin J. (2017). Medical service use and usual care of common shoulder disorders in Korea: a cross-sectional study using the Health Insurance Review and Assessment Service National Patient Sample. *BMJ Open*.

[14] Gill J. M., Mainous A. G. (1998). The Role of Provider Continuity in Preventing Hospitalizations. *Archives of Family Medicine*.

[15] Yelin E., Callahan L. F. (1995). Special article the economic cost and social and psychological impact of musculoskeletal conditions. *Arthritis & Rheumatism*.

[16] Weiss L. J., Blustein J. (1996). Faithful patients: The effect of long-term physician-patient relationships on the costs and use of health care by older Americans. *American Journal of Public Health*.

[17] McLeod J., McMurray J., Walker J. D., Heckman G. A., Stolee P. (2011). Care transitions for older patients with musculoskeletal disorders: continuity from the providers’ perspective. *International Journal of Integrated Care*.

[18] Kim L., Kim J.-A., Kim S. (2014). A guide for the utilization of Health Insurance Review and Assessment Service National Patient Samples. *Epidemiology and Health*.

[19] Felson D. T., Naimark A., Anderson J., Kazis L., Castelli W., Meenan R. F. (1987). The prevalence of knee osteoarthritis in the elderly. The Framingham Osteoarthritis Study. *Arthritis & Rheumatism*.

[20] Tellnes G., Bjerkedal T. (1989). Epidemiology of sickness certification - A methodological approach based on a study from Buskerud county in Norway. *Scandinavian Journal of Social Medicine*.

[21] Sundararajan V., Henderson T., Perry C., Muggivan A., Quan H., Ghali W. A. (2004). New ICD-10 version of the Charlson comorbidity index predicted in-hospital mortality. *Journal of Clinical Epidemiology*.

[22] Quan H., Sundararajan V., Halfon P. (2005). Coding algorithms for defining comorbidities in ICD-9-CM and ICD-10 administrative data. *Medical Care*.

[23] Sloane P., Egelhoff C. (1983). The relationship of continuity of care to age, sex, and race. *Journal of Family Practice*.

[24] Fleming M. F., Bentz E. J., Shahady E. J., Abrantes A., Bolick C. (1986). Effect of case mix on provider continuity. *Journal of Family Practice*.

[25] Lawrence R. C., Helmick C. G., Arnett F. C. (1998). Estimates of the prevalence of arthritis and selected musculoskeletal disorders in the United States. *Arthritis & Rheumatology*.

[26] Kim J., Son M. National Health Insurance Statistical Yearbook. Seoul: Health Insurance Review and Assessment Service and National Health Insurance Service.

[27] Kim H.-A., Kim S., Seo Y. I. (2008). The epidemiology of total knee replacement in South Korea: national registry data. *Rheumatology*.

[28] Kellgren J. H., Lawrence J. S. (1957). Radiological assessment of osteo-arthrosis. *Annals of the Rheumatic Diseases*.

[29] Woolf A. D., Pfleger B. (2003). Burden of major musculoskeletal conditions. *Bulletin of the World Health Organization*.

[30] Han K., Ha I., Lee J. (2017). Application of Health Care Big data and Necessity of Traditional Korean Medicine Data Registry. *Journal of Korean Medicine for Obesity Research*.

[31] Kang X., Fransen M., Zhang Y. (2009). The high prevalence of knee osteoarthritis in a rural chinese population: the wuchuan osteoarthritis study. *Arthritis Care Research*.

[32] Kim I., Kim H. A., Seo Y.-I., Song Y. W., Jeong J.-Y., Kim D. H. (2010). The prevalence of knee osteoarthritis in elderly community residents in Korea. *Journal of Korean Medical Science*.

[33] Blagojevic M., Jinks C., Jeffery A., Jordan K. P. (2010). Risk factors for onset of osteoarthritis of the knee in older adults: a systematic review and meta-analysis. *Osteoarthritis and Cartilage*.

[34] Choi Y.-J., Kang S.-H., Kim Y.-I. (2008). Association of higher continuity of primary care with lower risk of hospitalization among children and adolescent patients. *Korean Journal of Health Policy and Administration*.

[35] Gill J. M., Mainous 3rd A. G., Diamond J. J., Lenhard M. J. (2003). Impact of provider continuity on quality of care for persons with diabetes mellitus. *Annals of Family Medicine*.

[36] Christakis D. A., Mell L., Koepsell T. D., Zimmerman F. J., Connell F. A. (2001). Association of lower continuity of care with greater risk of emergency department use and hospitalization in children. *Pediatrics*.

[37] Sudo A., Miyamoto N., Horikawa K. (2008). Prevalence and risk factors for knee osteoarthritis in elderly Japanese men and women. *Journal of Orthopaedic Science*.

[38] Zhang Y., Xu L., Nevitt M. C. (2001). “Comparison of the prevalence of knee osteoarthritis between the elderly Chinese population in Beijing and whites in the United States: The Beijing Osteoarthritis Study. *Arthritis & Rheumatology*.

[39] Du H., Chen S.-L., Bao C.-D. (2005). Prevalence and risk factors of knee osteoarthritis in Huang-Pu District, Shanghai, China. *Rheumatology International*.

[40] Kim J. Basis of using health insurance data, strategic, and assignment from the computation of health statistic.

[41] Woolf A. D. (2000). The bone and joint decade 2000-2010. *Annals of the Rheumatic Diseases*.

[42] Cross M., Smith E., Hoy D. (2014). The global burden of hip and knee osteoarthritis: estimates from the global burden of disease 2010 study. *Annals of the Rheumatic Diseases*.

[43] Hong J. S., Kang H. C., Kim J. (2010). Continuity of care for elderly patients with diabetes mellitus, hypertension, asthma, and chronic obstructive pulmonary disease in Korea. *Journal of Korean Medical Science*.

[44] Sokol M. C., McGuigan K. A., Verbrugge R. R., Epstein R. S. (2005). Impact of medication adherence on hospitalization risk and healthcare cost. *Medical Care*.

[45] Peat G., McCarney R., Croft P. (2001). Knee pain and osteoarthritis in older adults: a review of community burden and current use of primary health care. *Annals of the Rheumatic Diseases*.

[46] Song Y. J. (2009). The south korean health care system. *Japan Medical Association Journal*.

[47] Yoon C.-H., Lee S.-J., Choo S., Moon O.-R., Park J.-H. (2007). Continuity of care of patient with diabetes and its affecting factors in Korea. *Journal of Preventive Medicine & Public Health*.

[48] Hong J., Kim J., Kang H. (2009). Continuity of Ambulatory Care among Adult Patients with Type 2 Diabetes and Its Associated Factors in Korea. *Korean Journal of Health Policy and Administration*.

